# Integrative Phosphoproteomics Links IL-23R Signaling with Metabolic Adaptation in Lymphocytes

**DOI:** 10.1038/srep24491

**Published:** 2016-04-15

**Authors:** Corinne Lochmatter, Roman Fischer, Philip D. Charles, Zhanru Yu, Fiona Powrie, Benedikt M. Kessler

**Affiliations:** 1 Kennedy Institute, Nuffield Department of Orthopedics Research Medical Science, Roosevelt Drive, Oxford OX3 7LF, UK; 2 Ludwig Institute for Cancer Research Ltd, Nuffield Department of Medicine, University of Oxford, Old Road Campus Research Building, Oxford OX3 7DQ, UK; 3 Target Discovery Institute, Nuffield Department of Medicine, University of Oxford, Roosevelt Drive, Oxford OX3 7FZ, UK

## Abstract

Interleukin (IL)-23 mediated signal transduction represents a major molecular mechanism underlying the pathology of inflammatory bowel disease, Crohn’s disease and ulcerative colitis. In addition, emerging evidence supports the role of IL-23-driven Th17 cells in inflammation. Components of the IL-23 signaling pathway, such as IL-23R, JAK2 and STAT3, have been characterized, but elements unique to this network as compared to other interleukins have not been readily explored. In this study, we have undertaken an integrative phosphoproteomics approach to better characterise downstream signaling events. To this end, we performed and compared phosphopeptide and phosphoprotein enrichment methodologies after activation of T lymphocytes by IL-23. We demonstrate the complementary nature of the two phosphoenrichment approaches by maximizing the capture of phosphorylation events. A total of 8202 unique phosphopeptides, and 4317 unique proteins were identified, amongst which STAT3, PKM2, CDK6 and LASP-1 showed induction of specific phosphorylation not readily observed after IL-2 stimulation. Interestingly, quantitative analysis revealed predominant phosphorylation of pre-existing STAT3 nuclear subsets in addition to translocation of phosphorylated STAT3 within 30 min after IL-23 stimulation. After IL-23R activation, a small subset of PKM2 also translocates to the nucleus and may contribute to STAT3 phosphorylation, suggesting multiple cellular responses including metabolic adaptation.

Molecular signaling cascades in normal physiology or disease mechanisms involve critical protein phosphorylation steps. Technological advancements in mass spectrometry have resulted in mapping >150000 protein phosphorylation sites^1^, but the physiological significance of most identified phosphorylation sites remains to be determined. The minority of them, in particular tyrosine phosphorylation, might be functionally relevant whereas the rest could simply be off-target reactions of activated kinases^2^,^3^. Phosphoproteomics employs selective phosphopeptide enrichment via immobilized metal affinity chromatography (IMAC) in general^4^,^5^,^6^, and titanium dioxide (TiO_2_) in particular^7^,^8^,^9^. Combining IMAC/TiO_2_ with hydrophilic interaction liquid chromatography (HILIC) improves recovery of phosphorylated peptides^10^,^11^,^12^. Combined phosphoenrichment strategies coupled to quantitative mass spectrometry such as isotope-labelling strategies^13^,^14^,^15^,^16^, led to deeper phosphorylation coverage of cellular signaling processes and subcellular compartments in cell lines and primary cells^17^,^18^. For instance, activation of lymphocytes by the T cell receptor (TCR) and cytokines such as IL-2 and IL-15 have been extensively mapped by phosphoproteomic approaches^14^,^15^,^19^,^20^,^21^,^22^,^23^. However, little is known about the molecular details of signaling after stimulation with less studied cytokines such as IL-23, which plays a key role in controlling innate and T-cell-mediated intestinal inflammation, thereby contributing to the pathology of inflammatory bowel disease (IBD), Crohn’s disease (CD) and ulcerative colitis (UC)^24^,^25^. Innate lymphoid and T cells are mainly driven by IL-23 to cause intestinal inflammation^26^,^27^,^28^. IL-23 dependent pathogenesis of autoimmunity is caused by the activation of a T helper cell subset (T_H_) producing a cytokine signature including IL-17, IL-6, GM-CSF, TNF, IFNγ, CCL20, IL1β and IL-23, thereby classifying them as T helper (T_H_17) cells^29^. IL-1β, IL-6, IL-23 and in some instances TGFβ are necessary for optimal T_H_17 differentiation, whereas IL-23 seems to be critical for the maintenance of the T_H_17 phenotype. The molecular nature of how lymphoid cells are activated beyond canonical JAK STAT signaling through the IL-23R is not yet well understood, but genetic association studies in IBD suggested links between JAK2, STAT3 and CCR6 gene polymorphisms and IL-23 signaling^30^. Also, impaired function of T_regs_ in patients with psoriasis is mediated by phosphorylation of STAT3 following stimulation with IL-23, IL-6 and IL-21^31^. Features distinguishing IL-23 from stimulation patterns of IL-6 and IL-21 as well as other related cytokines such as IL-2 and IL-12 that act through STAT3 are not yet clear. A number of downstream signaling molecules and pathways may be shared or part of the integrative networks. As IL-23 represents such a critical cytokine relevant for the development of pathogenic T_H_17 cells, we have applied a complementary set of quantitative phosphoproteomics based on enrichment strategies at the peptide or protein level to reveal comprehensive phosphorylation dynamics after IL-23 stimulation.

## Experimental Section

### Experimental design and statistical rationale

Phosphopeptide enrichment after 30 min of IL-23 stimulation was performed in three biological replicates. Unstimulated cells for each replicate were used as control (“0 minute” time point). Isotope labelled, fractionated phosphopeptides (HILIC/IMAC, 10 fractions per experiment) were analyzed by mass spectrometry after adjusting injection volumes to maximize peptide identification. Label-free phosphoprotein enrichment was performed as a time-course at 0 (CTRL), 5, 10 and 30 minutes after IL-23 stimulation in one single biological experiment, followed by mass spectrometry in analytical duplicates. The time resolved experimental design was employed to identify early responders and also to match with subsequent validation experiments using immunoblotting. Analytical duplicates were employed to increase the number of identified proteins and to enhance robustness of the analytical and quantitation workflows. Validation of candidate proteins was performed by immunoblotting in two additional biological replicates. STAT3 immunoprecipitations were performed as two independent biological experiments. Statistical methods and underlying rationales, in particular for the comparison of phosphopeptide versus phosphoprotein based enrichments, are described extensively in the data analysis and supplemental material sections.

### Cytokine stimulation

Kit225 cells were rested in medium without IL-2 at a cell density of 1 × 10^6^ cells/ml for 46 h before cytokine stimulation. Cells were then washed once in medium, counted, resuspended at 5 × 10^6^ cells/ml and incubated for another 2 h in order to rest them before they were spun down, resuspended in RPMI medium at the same cell density and divided up for the different conditions of cytokine stimulation. For all phosphoprotein experiments with different time points between 0–30 min, cells were stimulated at a cell density of 5 × 10^6^ cells/ml with 10 ng/ml IL-23 for the indicated amounts of time. Unstimulated control cells were collected at the same time. Phosphopeptide enrichment experiments were performed in biological triplicates Supplementary Methods. Two batches of cells were resuspended in 20 ml RPMI (experiments A and B –100 × 10^6^ cells/time point; 10 ml for experiment C –50 × 10^6^ cells/time point) and were stimulated either with IL-23 (30 min IL-23) by shaking at 37 °C (100 rpm), or without cytokine (0 min, control). For phosphopeptide experiments, cells were then pelleted at 4 °C and lysed in 100 μl lysis buffer (per 50 × 10^6)^ cells consisting of 10 mM sodium phosphate, pH 7.4 containing 150 mM NaCl, 1 mM EDTA, 0.5% NP-40, 0.1% sodium deoxycholate and 50 mM DTT+ protease inhibitors (Halt Protease Inhibitor Cocktail, Thermo Scientific) and phosphatase inhibitors (PhosSTOP Phosphatase Inhibitor, Roche), 1 mM sodium orthovanadate +2 mM beta-glycerophosphate, 10 mM NaF). Cells were lysed on ice for 20 min and insoluble material was pelleted by centrifugation at 4 °C before downstream processing of the lysate and phosphopeptide enrichment as described in the following section. Sample processing for phosphoprotein enrichment and subsequent validation experiments were performed as described below.

### Phosphopeptide enrichment

After clearing of the lysate by centrifugation at 14000 rpm, protein concentration was measured (BCA protein assay, Thermo Scientific) and adjusted between stimulated and unstimulated sample. Phosphorylation of STAT3 was tested at this stage by Western Blot using a small aliquot of lysate. Equal amounts of lysate (experiment A/B: 3.25 mg, C: 1.5 mg) were alkylated using 100 mM iodoacetamide (IAA) followed by chloroform/methanol precipitation as described previously^32^. Samples were resuspended in 200 μl 8 M urea, 25 mM TEAB, 0.8% N-octyl-glucopuranoside. Solubilized samples were diluted 8-fold with 25 mM TEAB and digested with trypsin (KR not P) overnight at 37 ^o^C. Samples were acidified with trifluoroacetic acid (TFA) and labeled on column with dimethyl- and [^2^H]_4_ dimethyl-formamide as described before^33^. Samples were pooled after elution, dried down and resuspended in 120 μl 80% acetonitrile (ACN)/0.1% TFA by first adding a final volume of 66% ACN/0.1% TFA followed by adjusting ACN concentration to 80%.

Phosphopeptides were enriched in a 2-step procedure (hydrophilic interaction chromatography (HILIC) followed by ion metal chromatography (IMAC) as described^12^. In brief, a HILIC column (Amide 80, 4.6 × 250 mm, Tosoh Bioscience LLC) was used to enrich and separate hydrophilic peptides into 30 fractions between 10 and 70 minutes on a Dionex 3000 UHPLC system. To accommodate the decreased solubility after dimethylation, peptides were loaded in 80% ACN. To reduce sample numbers, 1 ml peptide fractions eluted after HILIC chromatography were pooled (fractions 1–2, 3–4, 5–6, 7–8, 9–10, 11–13, 14–16, 17–21, 22–25, 26–30). The resulting 10 pooled fractions were incubated each with 30 μl of a 50% slurry of PHOS-Select Iron affinity gel (Sigma) for 30 min at RT and placed onto 0.22 μm nylon Spin-X centrifuge tubes for subsequent purification. Samples were washed two times with 0.5 ml 250 mM acetic acid/30% ACN followed by ddH_2_0 and eluted with 100 μl 400 mM ammonium hydroxide. Samples were lyophilized to dryness and kept at −80 ^o^C until analysis. Unbound material (flow through) in experiments A, B was dried down and stored at −80 ^o^C for subsequent TiO_2_ enrichment.

TiO_2_ enrichment was performed as described before with some modifications for single steps^34^,^35^. In brief, samples were resuspended in 120 μl GA solution (80 mg/ml glycolic acid/80% ACN/2% TFA). TiO_2_ beads (Titansphere 10 μm loose beads, Hichrom Ltd, UK) were washed with 0.6% NH_4_OH, then equilibrated with GA solution. Samples were incubated with 25 mg beads for 1.5 h shaking at RT and washed resuspending beads by vortexing followed by a centrifugation step at 8000 rpm. Washing steps were GA solution, 80% ACN/0.2% TFA and 20% ACN/0.5% TFA. Two elution steps were performed, first with 0.6% NH_4_OH, 1 h RT and then 60% ACN/0.05% TFA, 30 min RT collecting eluted peptides by centrifugation at 9000 rpm and followed by clearing over a C8 tip to remove residual beads^36^. Samples were dried down and stored at −80 ^o^C until analysis.

### Phosphoprotein enrichment

We performed three independent biological experiments stimulating 50 × 10^6^ cells Kit225 cells per time point with IL-23 or IL-2 (10 ng and 20 ng (360 U)/5 × 10^6^ cells) (experiments 1–3). Stimulation was in analogy to as described for phosphopeptide enrichment involving sequential addition of cytokine for shorter time points whilst cells were shaking in order to be able to stop all conditions at the same time. Samples from experiment 1 were used for analysis by mass spectrometry and validation by Western Blot, samples from experiment 2 and 3 for analysis by Western Blot only. Phosphoproteins were enriched using the Pierce Phosphoprotein enrichment Kit (Thermo Scientific, Pierce). In brief, after cytokine stimulation cells were washed twice in 10 ml cold 25 mM HEPES buffer before lysing cells in 1.2 ml lysis buffer (lysis/binding wash buffer with 0.25% CHAPS) containing protease inhibitors (Halt Protease Inhibitor Cocktail, Thermo Scientific) and phosphatase inhibitors (PhosSTOP Phosphatase Inhibitor, Roche) for 45 min on ice vortexing periodically. Lysates were then centrifuged at 3000 rpm for 30 mins at 4 ^o^C to remove cellular debris and pellet nuclei. Supernatant was collected and protein concentration was measured using BCA protein assay (Thermo Scientific), adjusted to 0.5 mg/ml with lysis buffer and 1 ml sample was then used per condition for phosphoprotein enrichment. Samples were loaded onto equilibrated IMAC resin cartridges, incubated for 30 min shaking at 4 ^o^C and washed 3× with 5 ml lysis/binding/wash buffer. Samples were eluted in elution buffer without CHAPS and concentrated as instructed. Protein concentration was measured and equal amounts (50 μg per sample) were reduced by adding 10 mM dithiothreitol (DTT), alkylated and subjected to chloroform-methanol precipitation, in-solution trypsin digestion and LC-MS/MS analysis as described in Supplementary Methods.

### Label-free quantitation (phosphoprotein and immunoprecipitation analysis)

**Figure 1:**
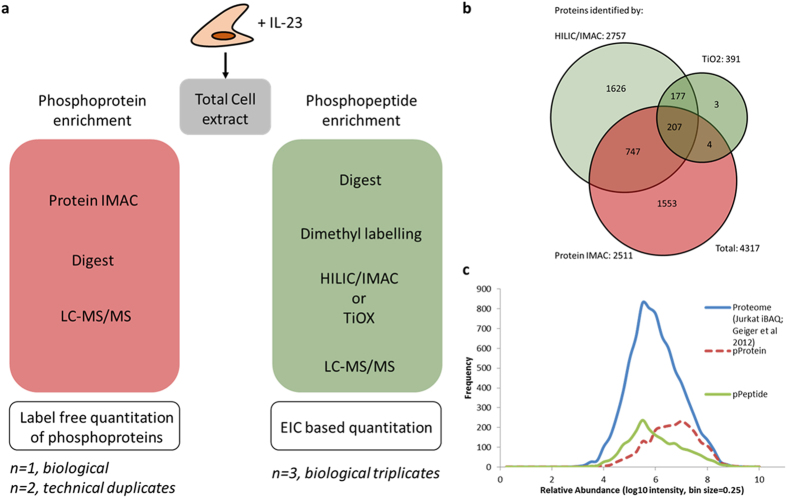
Comparison of phosphoprotein enrichment and phosphopeptide enrichment strategies. (**a**) Workflow for phosphoproteomic analysis of IL-23R signaling on protein and peptide level. Experimental repeats refer to proteomics discovery experiments only, and validation experiments were performed independently. (**b**) Proteins identified following phosphopeptide and phosphoprotein enrichment share only limited overlap, demonstrating high complementarity of the 2 approaches. (**c**) The relative abundance profile of identified proteins by phosphopeptides (IMAC peptides only) and phosphoproteins as compared to the whole proteome demonstrate higher sensitivity of the phosphopeptide approach.

We used label-free quantitation with Progenesis QI for proteomics (QIP) v2.0 to determine differentially phosphorylated proteins after phosphoprotein enrichment and a time course stimulation with IL-23 for 0, 5, 10, 15 and 30 minutes. Samples of each time point were analysed in technical duplicates. Proteins were identified with Mascot v2.5 (10 ppm precursor and 0.05 Da fragment mass tolerance, UniProt Swiss-Prot human database (retrieved 08/12/2013), 1% FDR, peptide score threshold of 20). We used all significantly identified peptides to normalize the data and unique peptides for quantitation. Quantification of STAT3 derived peptides following immunoprecipitation in cytosolic and nuclear fractions was performed by separate label-free quantification using LC-Progenesis QI software, essentially as described above. In this case, no normalization step was performed to reflect isolated STAT3 protein levels in the analysed equal amounts of subcellular compartments post IL-23 stimulation. For the purposes of comparing the numbers of phosphoproteins identified between enrichment approaches in Fig. 1, an export of the LC-Progenesis QI dataset output was re-analysed through Proteome Discoverer (PD) v1.4 (Thermo Scientific) as described above for the phosphopeptide enrichment. This was to ensure that the same protein grouping algorithm was applied for both datasets.

Further information about i) Cell lines and antibodies, ii) Analysis by tandem mass spectrometry, iii) Data analysis, iv) Statistical analysis of differential phosphorylation at the peptide & protein level, v) Subcellular fractionation, immunoprecipitation and immunoblotting, vi) Gene expression analysis, and vii) Lactate measurement by GCxGC-MS is available in supplemental information.

## Results

### Complementary nature of phosphoprotein and phosphopeptide enrichment

We first set out to compare phosphorylation enrichment protocols based on the protein or peptide level. As a study case, initial phosphorylation-dependent signaling events in T cells after IL-23 stimulation were examined. To this end, Kit225 lymphocytic cells were treated with IL-23 followed by either peptide (0 and 30 minutes IL-23 stimulation) - or protein (0, 5, 10 and 30 minutes IL-23 stimulation) - based phospho-enrichments and subsequent mass spectrometric (MS) analysis (Fig. 1a). After phosphopeptide enrichment with HILIC/IMAC, we were able to identify a total of 7,956 unique phosphopeptides (Table S1, unique combination of sequence and phosphorylated residues) in HILIC/IMAC fractions from 111095 peptide spectrum matches (PSMs) (assigning matches to 19.3% of total spectra acquired) representing 2757 unique phosphorylated proteins (Fig. 1b). Phospho-PSMs represented up to 80% of total PSMs in individual HILIC/IMAC fractions across all three enrichments. Further TiO_2_ enrichment of the unbound material (yielding 534 unique phosphopeptides) raised total unique phosphopeptides identified to 8069 (representing 2764 unique phosphorylated proteins). The phosphoprotein enrichment approach revealed a total of 2511 unique proteins, including 116 proteins with direct phosphopeptide evidence (Table S2; for this group of proteins, we did not observe corresponding phosphopeptides in all cases, so we refer to them as phosphoenriched proteins). 221 unique phosphopeptides were identified from the phosphoprotein enrichment, further raising total of uniquely identified phosphopeptides to 8202 when added to those from the phosphopeptide enrichment. Phosphopeptide and phosphoprotein enrichment together identified a combined total of 4317 unique proteins with only limited overlap (958 proteins, representing 22%) between phosphopeptide enrichment (HILIC/IMAC/TiO_2_), and phosphoprotein enrichment (Fig. 1b). To demonstrate the differential sensitivity of both methods, we then used iBAQ values, published by^37^, for the proteins identified with both approaches. The use of the human T-cell line Jurkat as reference and the comparison to a measured total cell lysate allows the estimation of the relative abundance of the detected proteins in both phosphoproteomic methods (Fig. 1c).

**Figure 2:**
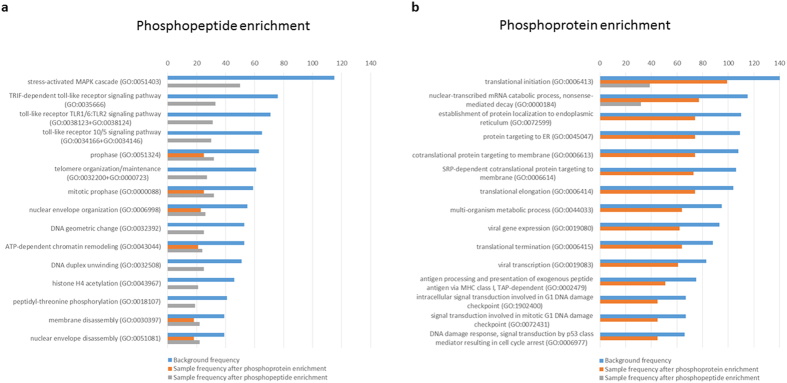
Gene Ontology (biological process) term enrichment analysis. We extracted gene ontology terms (and their background frequency - blue bars) for the lists of identified proteins following phosphopeptide enrichment and phosphoprotein enrichment. The top 15 pathways are shown. (**a**) We identified 50 of 115 proteins in “stress-activated MAPK cascade (GO:0051403) and several TLR associated signaling pathways (grey bars). Several of the 15 best covered GO terms are also covered by phosphoprotein enrichment (orange bars). (**b**) Proteins identified following phosphoprotein enrichment cover other biological processes than following phosphopeptide enrichment, highlighting the complementary nature of the two phosphoproteomic approaches.

In accordance with the limited overlap of proteins and different sensitivity between both phosphoproteomic approaches, we observed that different cellular functions are enriched after gene ontology analysis (GO, gene_ontology_edit.obo.2014-12-01) of identified phospho-enriched proteins (Fig. 2). The peptide-based method enriched for protein expression profiles associated with MAP kinase signaling or Toll-like receptor signaling, whereas phosphoprotein enrichment led to signatures related to basic cellular mechanisms such as DNA damage repair and translation. It is of note that we observed almost no overlap in the 15 most enriched cellular functions between the phosphoproteomic approaches.

We applied several statistical approaches to delineate relevant phosphorylated proteins and phosphosites that were found to be either up- or down-regulated after IL-23 stimulation (Fig. S1). For instance, we found that a p-value approach (volcano plots) appear to be more reliable for phosphopeptides (Fig. S1a,b), whereas a soft clustering approach (Mfuzz) considering all measured time points was more accurately describing changes in phosphoproteins (Fig. S1d–f). Principal component analysis (PCA) (Fig. S1c) showed that variance in protein expression profiles had little correlation with significantly changing phosphopeptides. Proteins for which alterations were observed at the protein as well as specific phosphosite levels are summarized in Tables 1 and S3).

### IL-23 triggers STAT3 phosphorylation and nuclear translocation

**Figure 3:**
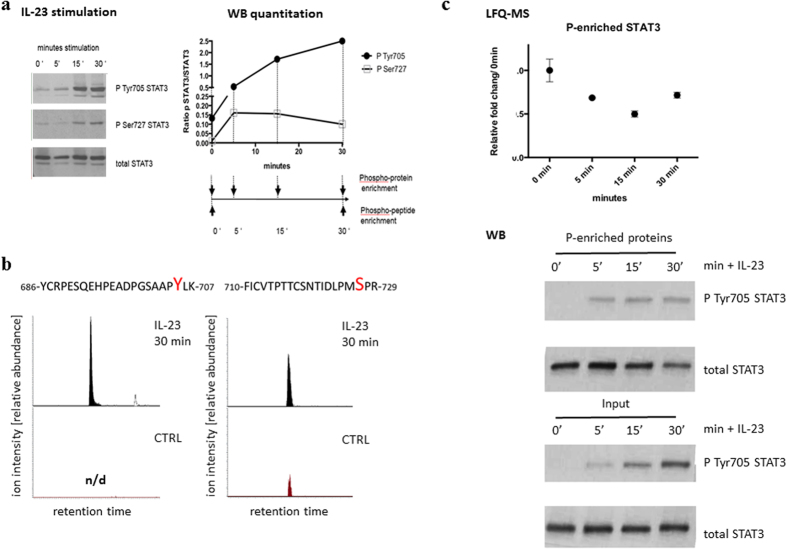
Activation of STAT3 signaling in Kit225 cells stimulated with IL-23. (**a**) Kinetics of Tyr705 and Ser727 STAT3 phosphorylation (whole cell lysate) in response to IL-23 stimulation. Western Blot and quantitation of pTyr705 STAT3/STAT3 and pSer727 STAT3/STAT3 (normalized to total STAT3 levels) are shown. (**b**) Relative abundance of pTyr705 and pSer727 STAT3 peptides as detected by LC-MS (extracted ion chromatograms). (**c**) Levels of total STAT3 detected in soluble phosphoenriched (P-enriched) protein fractions after IL-23 stimulation as measured by label-free quantitation MS (LFQ-MS: quantitation is based on all STAT3 derived peptides identified in two technical duplicates, top panel). pTyr705 STAT3 and total STAT3 levels detected by Western Blot (bottom panel) before (Input) and after phosphoenrichment (P-enriched).

As an initial validation experiment, Kit225 cells known to respond to IL-12 stimulation^38^ and expressing the IL-23R subunit (Fig. S2) were stimulated with recombinant human IL-23, and a time-dependent increase in phosphorylated STAT3 at the positions pTyr705 and pSer727 was observed (Fig. 3a). Using tandem mass spectrometry, we were able to monitor an increase in phosphorylation on those sites after 30 minutes of stimulation on the peptide level as quantified by differences in dimethyl labelled peptides and comparing their integrated MS spectra between samples (Fig. 3b). The pSer727 STAT3 site was detected in all three (datasets A, B and C) and the pTyr705 STAT3 site in the two datasets where an input of ~10^8^ cells starting material was used per time point (datasets A and B). To complement the phosphopeptide data, we performed phosphoprotein enrichment after simulating Kit225 cells with IL-23 for the indicated amount of times and were analyzing total protein levels in the phosphoenriched samples using label-free quantitative (LFQ) MS. Interestingly, we observed a decrease in total STAT3 levels over time after IL-23 stimulation (Fig. 3c top panel). We were able to confirm this result in three independent biological replicates by blotting for total STAT3 in the phosphoprotein enriched fractions (Fig. 3c, Supplemental Fig. S3a). Stimulation of Kit225 cells with recombinant IL-2 resulted in an increase in pTyr705 and pSer727 STAT3 (Fig. S3b). We also noted a decrease in total STAT3 levels after IL-2 stimulation in the phosphoprotein enrichment samples as quantified by MS and Western Blot (Fig. S3c,d).

**Figure 4:**
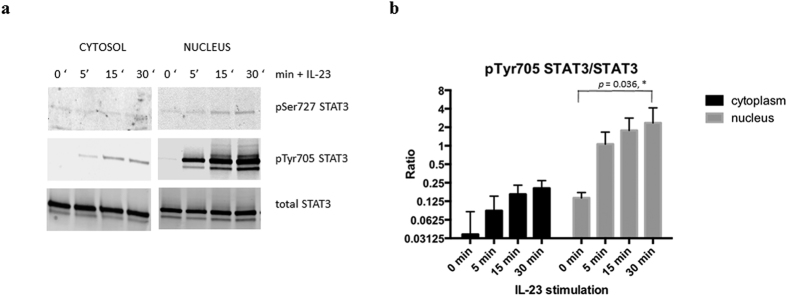
STAT3 phosphorylation in cytosol and nuclear enriched fractions in response to stimulation with IL-23. (**a**) Representative Western Blot of phosphorylated and total STAT3 in cytoplasmic and nuclear enriched protein fraction, (**b**) Quantitation of pTyr705 STAT3/STAT3 levels by Western Blot in cytoplasm (black) and nuclear enriched fraction (grey) in response to IL-23 stimulation, n = 3; p value indicated for comparison of nuclear pTyr705 STAT3/STAT3 levels between 0 and 30 minutes (paired t test using log-transformed data).

To investigate whether decrease in STAT3 levels after cytokine stimulation in the phosphoprotein enriched samples was due to a translocation to the nucleus, we stimulated Kit225 cells as before followed by separation of cytosolic and nuclear enriched protein fractions and blotting for phosphorylated and non-phosphorylated STAT3. We observed a rapid increase in phosphorylation of Tyr705 in the nuclear enriched fraction as compared to the cytosolic one after stimulation with IL-23 (Fig. 4a,b) which was much less pronounced in cells stimulated with IL-2 (Fig. S4a). Also, the phosphorylation increase for the site pTyr705 was stronger than for pSer727 (Figs 3a and 4a).

**Figure 5:**
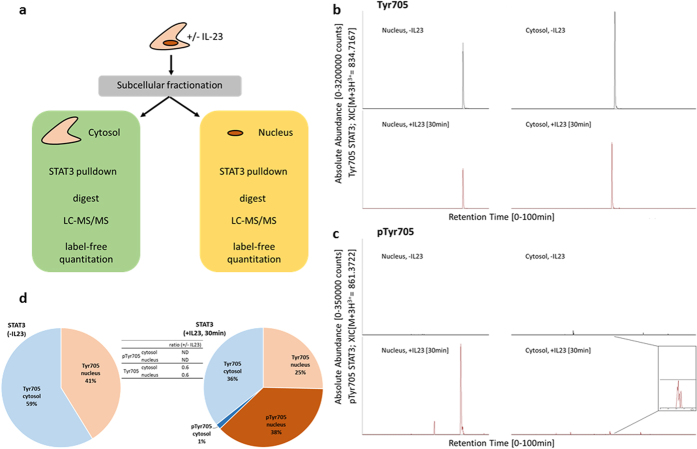
Immunoprecipitation of endogenous STAT3 and phosphosite analysis. (**a**) IP Workflow. (**b**,**c**) Extracted ion chromatograms (XIC) of nucleus/cytosol detected Tyr705 covering peptide of STAT3 in phosphorylated and non-phosphorylated form after IL-23 stimulation. pTyr705 is induced in the cytosol and nucleus after stimulation while the cytosol/nucleus ratio of the non-phosphorylated variant is not changed. However, the absolute amount of Tyr705 unphosphorylated STAT3 is decreased by the number of molecules phosphorylated after induction. The combined data allows the calculation of a correction factor for the differential ionisation efficiency of phosphorylated and unphosphorylated form of the peptide (Fig. S7). (**d**) We modelled the occupancy of pTyr705 in the two subcellular compartments depending on IL-23 stimulation after quantitation by XIC. As ionization efficiency can be different for the phosphorylated form of the Tyr705 covering peptide, we calculated a correction factor, assuming that the decrease in non-phosphosignal equals the gain of the phosphosignal, indicating a 4.3-fold lower ionization efficiency for the phosphorylated peptide. The application of this factor to measured intensities allows the calculation of the pTyr705 occupancy.

To further quantify the observed dynamics on Tyr705 STAT3 phosphorylation, we stimulated Kit225 cells with IL-23 for 30 min, separated cytosolic and nuclear fractions followed by immunoprecipitation of STAT3 and quantitation of phosphorylation sites by mass spectrometry (Fig. 5a). Protein sequence coverage of immunoprecipitated STAT3 was 80%, including a tryptic peptide fragment containing phosphorylated Tyr705 (Fig. 5b) enabling its quantitation. Analysis of STAT3 pTyr705 levels in the cytosol and nucleus before and after stimulation with IL-23 showed a drastic increase of pTyr705 in the nucleus, but phosphorylation of this residue was close to the limit of detection in the cytosol (Fig. 5b,c). To calculate the occupancy of pTyr705 depending on stimulation and subcellular localization of STAT3, we applied a correction factor to account for the difference in ionization efficiency^39^ between the phosphorylated peptide species and its non-phosphorylated counterpart. Assuming that the loss of detected signal-intensity of the Tyr705 peptide after stimulation should equal the gain of the pTyr705 signal, the difference in ionization efficiency can be inferred to be ~4.3-fold higher for the non-phosphorylated form as compared to its non-phosphorylated counterpart (Fig. S7). Based on this, we were able to determine the quantities of different STAT3 forms and their localization before and after stimulation. pTyr705 phosphorylation represents 38% of the total STAT3 material predominantly localized in the nucleus after 30 min stimulation with IL-23 (Fig. 5c).

Our MS results were confirmed by Western Blotting, showing that pTyr705 STAT3/STAT3 similarly increased in the nuclear fraction after 30 minutes of cytokine stimulation (Fig. S5c). There was no significant increase in phosphorylation of pSer727 in nuclear STAT3 between unstimulated and stimulated cells as opposed to what has been observed for the pTyr705 site (Fig. S5b,c). Together, we conclude that IL-23 leads to Tyr705 and Ser727 STAT3 phosphorylation and increased levels of pTyr705 STAT3 in the nucleus.

### Expanding the IL-23R phosphorylation cascade

**Figure 6:**
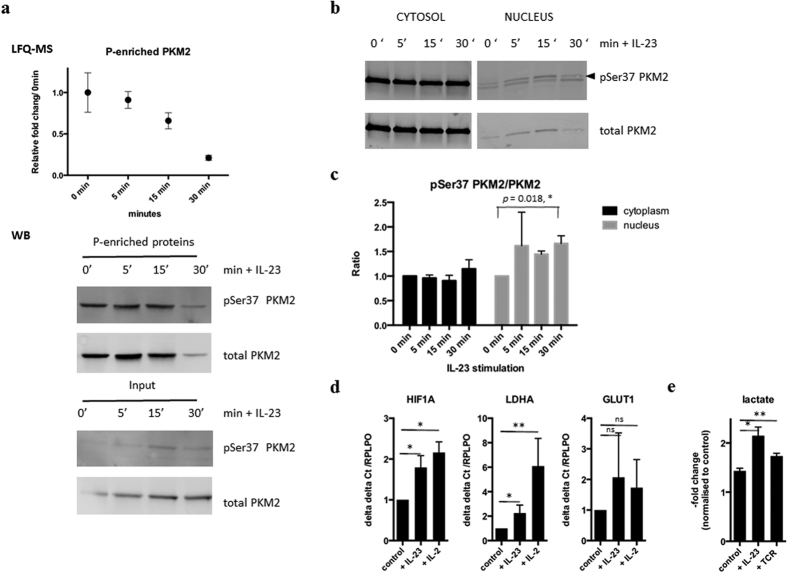
PKM2 phosphorylation, nuclear translocation and activation of glycolysis after IL-23 stimulation. (**a**) Levels of total PKM2 detected in phosphoenriched protein fractions after IL-23 stimulation measured by label free quantitation MS (LFQ-MS, top panel). pSer37 PKM2 and total PKM2 levels detected by Western Blot either before (Input) or after phosphoenrichment (P-enriched proteins). Representative Blots from experiment 1 are shown (bottom panel). (**b**) Representative Western Blot of phosphorylated and total PKM2 in cytoplasmic and nuclear enriched protein fractions. (**c**) Quantitation of pSer37 PKM2 and PKM2 levels shown as pSer37 PKM2/PKM2 ratios by Western Blot in cytoplasm (black) and nuclear enriched fraction (grey) in response to IL-23 stimulation, n = 3; p value indicated for comparison between nuclear pSer37 PKM2/PKM2 levels between 0 and 30 minutes (paired t test using log-transformed data). (**d**) HIF1A, LDHA and GLUT1 gene expression assessed by RT-PCR in Kit225 cells stimulated with IL-23 or IL-2 for 24 h vs. control (normalized to RPLPO levels, unstimulated cells rested for 24 h). Data represent means ± SD of 3 independent experiments; ^∗^p < 0.05, ^∗∗^p < 0.05 (paired t test using log-transformed data). (**e**) Intracellular lactate measured by GC-MS in cells treated with IL-23 or TCR stimulation for 24 h vs control (cells rested for 24 h). Values shown are mean ± standard deviations (SD) of n = 3 technical replicates and are normalized to basal levels at t = 0 h. ^∗^p < 0.05, 8 ^∗∗^p < 0.05 (unpaired t test).

Interrogation of our phosphoproteomic data for overlapping information between the phosphopeptide and phosphoprotein enrichment strategies revealed a number of novel proteins with induced site-specific phosphorylations which have not been described in the context of IL-23 signaling before. These include pyruvate kinase PKM2 (pSer37), lymphocyte specific protein LSP1 (pSer204), LIM and SH3 domain protein 1 LASP1 (pThr34) and Ataxin (pSer775) (Tables 1 and S3 and Fig. S1c). PKM2 was previously shown to phosphorylate STAT3 pTyr705 in the nucleus directly^40^, so we further examined total and phosphoprotein levels of PKM2 after IL-23 stimulation (Fig. 6 and Fig. S6).

### IL-23 triggers PKM2 phosphorylation, nuclear translocation and activates glycolysis

**Figure 7:**
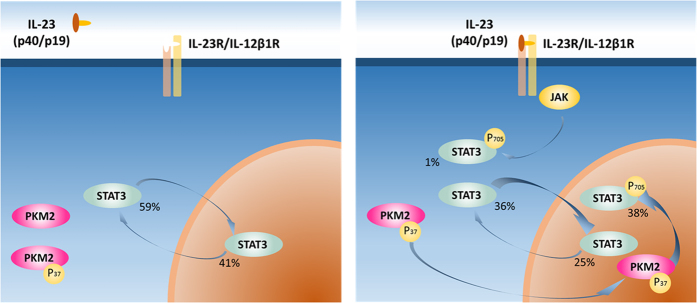
IL-23 triggers phosphorylation and nuclear translocation of STAT3 and PKM2. Phosphorylation events after IL-23 stimulation include STAT3 phosphorylation on Tyr705 by the kinases JAK in the cytosol^62^ but also other enzymes such as PKM2, a fraction of which also translocates to the nucleus. As the abundance of pSer37 PKM2 in the cytosol appears static (Fig. 6b), we hypothesize that a proportion of pSer37 PKM2 accumulates in the nucleus. Together with the data presented in Fig. 6a, these observations indicate the nuclear translocation of a fraction of PKM2. Quantitative mass spectrometry allowed the determination of the different STAT3 protein and phosphorylation stoichiometries within the cytosol and nucleus (Fig. 5b,c). STAT3 Tyr705 phosphorylation also increases after nuclear translocation, and kinases such as PKM2 may contribute to this signaling cascade.

Quantitation of PKM2 protein levels by mass spectrometry and immunoblotting in phosphoenriched protein fractions revealed a decrease after IL-23 stimulation as compared to total input (Fig. 6a). Analog to STAT3, we observed an accumulation of the phosphorylated form of PKM2 (pSer37) in nuclear enriched fractions as a response to IL-23 stimulation (Fig. 6b). The vast majority of STAT3 and PKM2 phosphorylation appears to occur within the nuclear compartment as assessed by immunoblotting (Figs 4 and 6) and quantitative mass spectrometry (Figs 6 and 7). These effects were not observed to the same extent when cells where stimulated with IL-2 (Figs S6a,b and S4c). To further validate effects of PKM2 downstream signaling, we analyzed glycolytic gene expression. There were significant changes in HIF1A (hypoxia inducible factor alpha) and LDH (lactate dehydogenase), but not GLUT1 (glucose transporter type 1) mRNA levels in Kit225 cells stimulated with IL-23 or IL-2 for 24 h as compared to control cells (Fig. 6d). In addition, we measured intracellular lactate levels in IL-23 or TCR stimulated Kit225 cells versus rested cells after 24 h by GCxGC-MS and found increased lactate levels in response to both stimuli (Fig. 6e). Neither IL-23 nor TCR stimulation in Kit225 cells result in cell proliferation in contrast to IL-2 stimulation. Taken together, these results expand the range of IL-23 induced signaling towards activation of glycolysis.

## Discussion

In our study, we compared the two different phosphoproteomic strategies and found that both methods are highly complementary in protein identifications and coverage of cellular functions (Figs 1 and 2). As most proteins in the cell become phosphorylated at any time during their lifetime, phosphoprotein enrichment may suffer more from the detection of off-target and background phosphorylation than at the phosphopeptide. As a consequence, phosphoenrichment on the protein level appears to detect proteins at the higher end of protein abundance in the cell, while proteins identified following phosphopeptide enrichment are generally less abundant. Therefore, integration of the two methods increases the range of phosphoproteome coverage (Fig. 1c). Another disadvantage of phospho-enrichment on the protein level is the method’s susceptibility to enrich proteins containing domains of highly enriched acidic amino acid residues and possible co-enrichment of protein interaction partners irrespective of phosphorylation status. It can be assumed that all proteins have a certain affinity to the IMAC resin which is then further increased by phosphorylation, resulting in a dilution effect of differentially observed phosphorylation events. At the protein level, quantitation of a phosphorylation event is supported by mostly multiple peptides rather than a single (phosphorylated) one. This is also beneficial for normalization as enriched (phosphorylated proteins) can be compared to the non-differential co-enrichment of non-specific binders, therefore advocating affinity enrichment rather than affinity purification^41^.

We applied a statistical approach based on the ‘significance A’ p-value method^42^ to both phosphopeptide and phosphoprotein enriched samples and found slightly different traits between differentially regulated phosphomaterial on the peptide versus protein level (Fig. S1a,b). This can be explained by the fact that phosphoproteins mostly contain multiple phosphorylation sites from which only one or a few are regulated during stimulation, hence affecting enrichment in a different fashion than at the peptide level. In addition, phosphorylation linked to subcellular translocation can further complicate such analyses as changes in phosphorylation at lower ratios may also be biologically relevant. To better reflect subcellular localization at a statistical level, we applied a soft clustering approach taking into account all time points measured during cytokine stimulation (Fig. S1c). This analysis showed very little overlap with the statistical method used to describe changes observed at the phosphopeptide level. However, soft clustering of protein expression profiles revealed multiple proteins that are translocated to the nucleus after cytokine stimulation, representing part of a wider signaling wave (Fig. S1d–f). For instance, next to PKM2 (Fig. S1f), we have also observed similar trends for other proteins such as CDK6, LSP1, LASP1 and OTUB1 in our MS experiments (Tables 1 and S3). Phosphorylation of OTUB1 on Ser16 has been implicated in nuclear targeting^43^. While pThr34 LASP-1 is a site that has not been reported previously, phosphorylation of LASP-1 on Ser146 we detected to be 1.4-fold increased upon IL-23 stimulation (Table 1) has been reported to induce nuclear translocation of LASP-1^44^. LSP-1 and CDK6, which have been shown to phosphorylate p65 in the nucleus, act as transcriptional co-activators inducing pro-inflammatory gene expression and are also phosphorylated, but this has not yet been described in the context of nuclear targeting^45^,^46^,^47^,^48^.

STAT3 becomes phosphorylated at multiple sites including Tyr705 and Ser727 by a variety of stimuli, followed by dimerization and translocation to the nucleus^49^. Interestingly, translocation has been shown to be tyrosine phosphorylation independent^50^. Our quantitative data sheds unprecedented details into this process showing that in rested T-cells, comparable numbers of STAT3 molecules reside in the cytosol (59%) and also in the nucleus (41%) (Figs 4 and 7). The levels of pTyr705 STAT3 in rested cells are not detectable (Fig. 5b). Upon stimulation with IL-23, a small proportion of cytosolic STAT3 becomes phosphorylated on Tyr705, while a notable proportion of nuclear STAT3 molecules (38%) become phosphorylated on Tyr705 after 30 minutes. Quantitation of the different STAT3 molecule populations suggest that phosphorylation of the nuclear portion occurs as well as nuclear translocation of phosphorylated species (Fig. 7). IL-23 induced a much higher level of additional pTyr705 STAT3 phosphorylation as compared to IL-2 (Fig. 4, Figs S3 and S4). This would suggest additional STAT3 phosphorylation events in the nucleus after and/or independent of translocation. Interestingly, PKM2 can phosphorylate STAT3 on Tyr705 in a JAK2, c-Src independent mechanism^40^. PKM2 was also identified as being associated with STAT3^51^. Of note is that we find c-Src at decreased levels in total phosphoprotein enrichment experiments (Table S2 and S3). A small subset of PKM2 translocates into the nucleus in response to EGFR stimulation upon pSer37 phosphorylation^52^. pSer37 PKM2 recruits PIN1 for cis-trans isomerization of PKM2, which promotes PKM2 binding to importin α5 and translocating to the nucleus. This has been shown to be Erk1/2 dependent and contribute to the Warburg effect^53^. Furthermore, PKM2 dimers promote cell proliferation, and PKM2 protein kinase activity is essential for tumor growth^40^. Our results would suggest a similar outcome after IL-23 stimulation in T cells. Not surprisingly, stimulating the IL-2 dependent Kit225 cell line with IL-2 resulted in proliferation and hence induction of glycolysis due to cell proliferation described for lymphocytes^54^, and we also observed the same pattern of HIF1A, LDH and GLUT1 gene induction after IL-23 stimulation without resulting in cell proliferation within 24 hours. Consistent with this, we confirmed lactate production in response to IL-23 using TCR stimulation as control. TCR based activation is known to induce lactate production via induction of GLUT-1 surface expression, promoting glucose uptake^55^,^56^. T cells under T_H_17 polarizing conditions undergo a HIF1-α dependent metabolic switch to glycolysis, and our data indicates that IL-23 might be contributing to this effect via PKM2 and HIF1-α^57^. IL-23 is also induced by lactate^58^, thereby possibly leading to a positive loop effect. HIF1-α induction has been associated to IL-23 in dendritic cells^59^ and a link between HIF1-α and PKM2 has been established in cancer cells^60^. PKM2 may therefore exert more than one function and next to its role in glycolysis, it can also activate transcription by acting as a protein kinase^40^. Taken together, IL-23, notably signaling through PKM2/STAT3, might contribute to the metabolic T cell phenotype of Th17 cells and, together with IL-6^61^, may constitute an essential factor for lineage commitment.

## Additional Information

**Data Availability**: The mass spectrometry proteomics data have been deposited to the ProteomeXchange Consortium via the PRIDE partner repository with the dataset identifier PXD002990 and 10.6019/PXD002990. 

**How to cite this article**: Lochmatter, C. *et al.* Integrative Phosphoproteomics Links IL-23R Signaling with Metabolic Adaptation in Lymphocytes. *Sci. Rep.* **6**, 24491; doi: 10.1038/srep24491 (2016).

## Supplementary Material

Supplementary Information

Supplementary Table S1

Supplementary Table S2

Supplementary Table S3

## Figures and Tables

**Table 1:** List of selected IL-23 downstream targets with regulated phosphorylation sites.

Peptide	Protein Accession	Protein Description	Start	End	Observed Phospho-sites	FOLD CHANGE* (P-peptide)	FOLD CHANGE** (P-protein)
WSAPESR	P54253	Ataxin-1	774	780	S775	4.7	2.4
LDIDSPPITAR	P14618	Pyruvate kinase PKM	33	43	S37	1.5	0.2
LIDRTESLNR	P33241	Lymphocyte-specific protein 1	198	207	S204	3.1	0.2
ACFHCETCK	Q14847	LIM and SH3 domain protein 1/LASP-1	28	36	T34	2.9	0.3

We list phosphorylated proteins detected in both, the phosphopeptide and phosphoprotein enrichment approaches, for which the most prevalent changes (up- or down-regulation) after IL-23 stimulation (0 versus 30min) were measured. The columns are showing phosphopeptides identified with corresponding protein accession number (UniProt), protein description, start and end amino acid position of identified phosphopeptide within the protein, quantitation represented by the phosphopeptide mass peak intensity ratio of stimulated/unstimulated conditions (∗ ) and relative protein abundance in whole phosphoprotein enriched fractions measured by labelfree quantitation MS for IL-23 stimulated samples (30min) normalized to unstimulated sample (0min) (∗∗).
