# Supplementary material for: Integrative Phosphoproteomics Links IL-23R Signaling with Metabolic Adaptation in Lymphocytes
Source: Sci Rep. 2016 Apr 15;6:24491. doi: 10.1038/srep24491 (PMC4832251; doi:10.1038/srep24491)
Supplement: Supplementary Information [file srep24491-s1.pdf]

Integrative Phosphoproteomics Links IL-23R Signaling with Metabolic Adaptation in Lymphocytes

[+,#*]*Corinne Lochmatter,* [+,$*]*Roman Fischer,* [$]*Philip D. Charles,* [$]*Zhanru Yu,* [+]*Fiona Powrie,* [$]*Benedikt M. Kessler*

[+]Kennedy Institute, Nuffield Department of Orthopedics Research Medical Science, Roosevelt Drive, Oxford OX3 7LF, UK

[#]Ludwig Institute for Cancer Research Ltd, Nuffield Department of Medicine, University of Oxford, Old Road Campus Research Building, Oxford OX3 7DQ, UK

[$]Target Discovery Institute, Nuffield Department of Medicine, University of Oxford, Roosevelt Drive, Oxford OX3 7FZ, UK

*equal contribution

SUPPLEMENTAL INFORMATION

SUPPLEMENTAL FIGURES

Figure S1. ***Distribution of quantitative phosphorylation data***

Figure S2. ***IL-23R expression in Kit223 cells***

Figure S3. ***Activation of STAT3 signaling in Kit225 cells stimulated with IL-23 and IL-2.***

Figure S4. ***IL-23 triggers phosphorylation of STAT3 predominantly in the nucleus***

Figure S5. ***STAT3 phosphorylation in the cytosol and nucleus***

Figure S6. ***Phosphorylation of PKM2 after IL-23 or IL-2 stimulation***

Figure S7. ***MS quantitation of STAT3 phosphorylation at Tyr705 after IL-23 stimulation***

SUPPLEMENTAL TABLES

Table S1. ***Phosphopeptides identified by mass spectrometry***

Table S2. ***Phosphoproteins identified by mass spectrometry***

Table S3. ***IL-23 altered downstream phosphoprotein targets with overlapping phosphosites identified***

SUPPLEMENTAL METHODS

Table S4. ***Experimental design of the phosphopeptide based proteomics approach used in this study***

SUPPLEMENTAL REFERENCES

SUPPLEMENTAL FIGURES

Figure S1

a

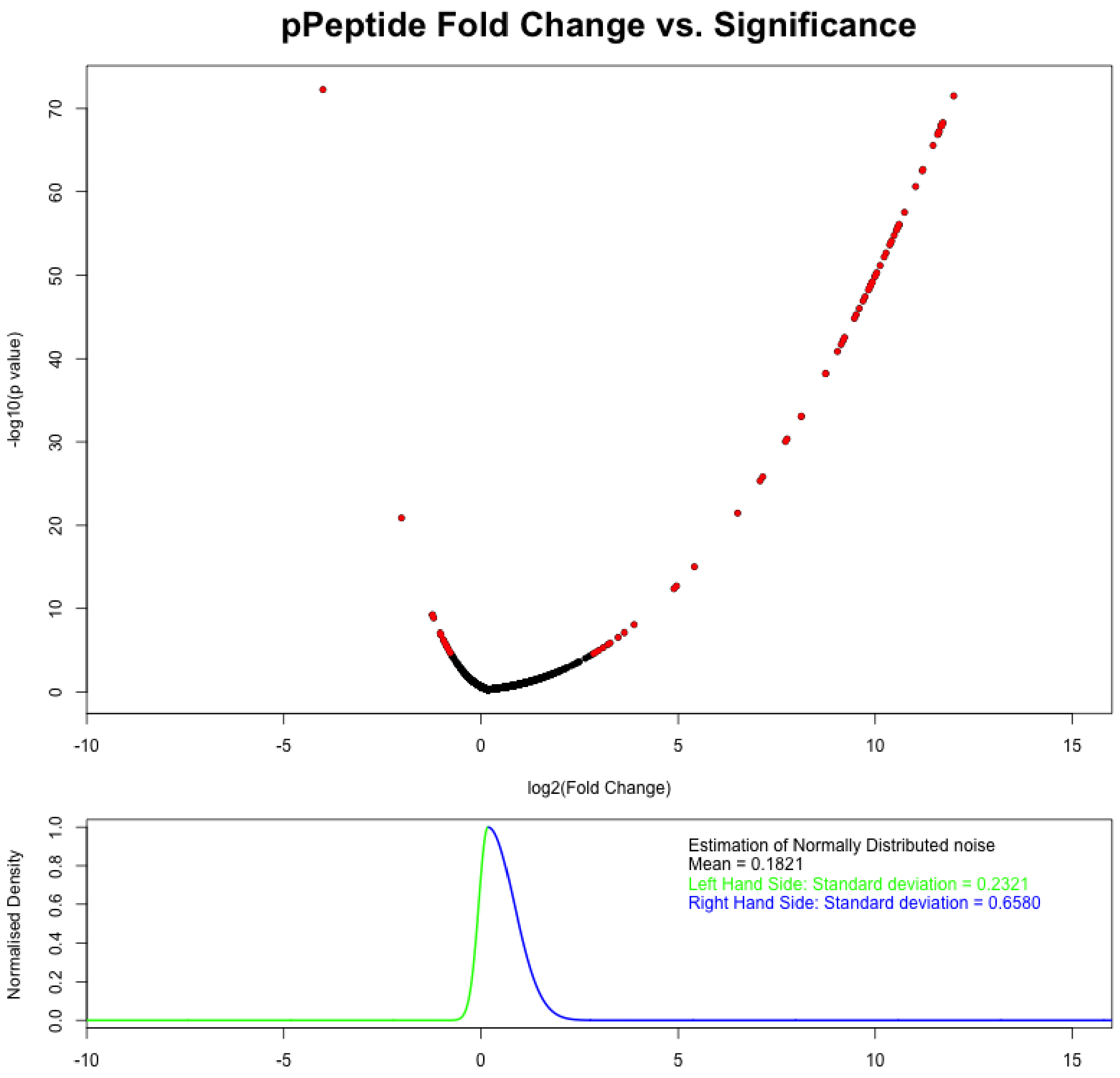

b

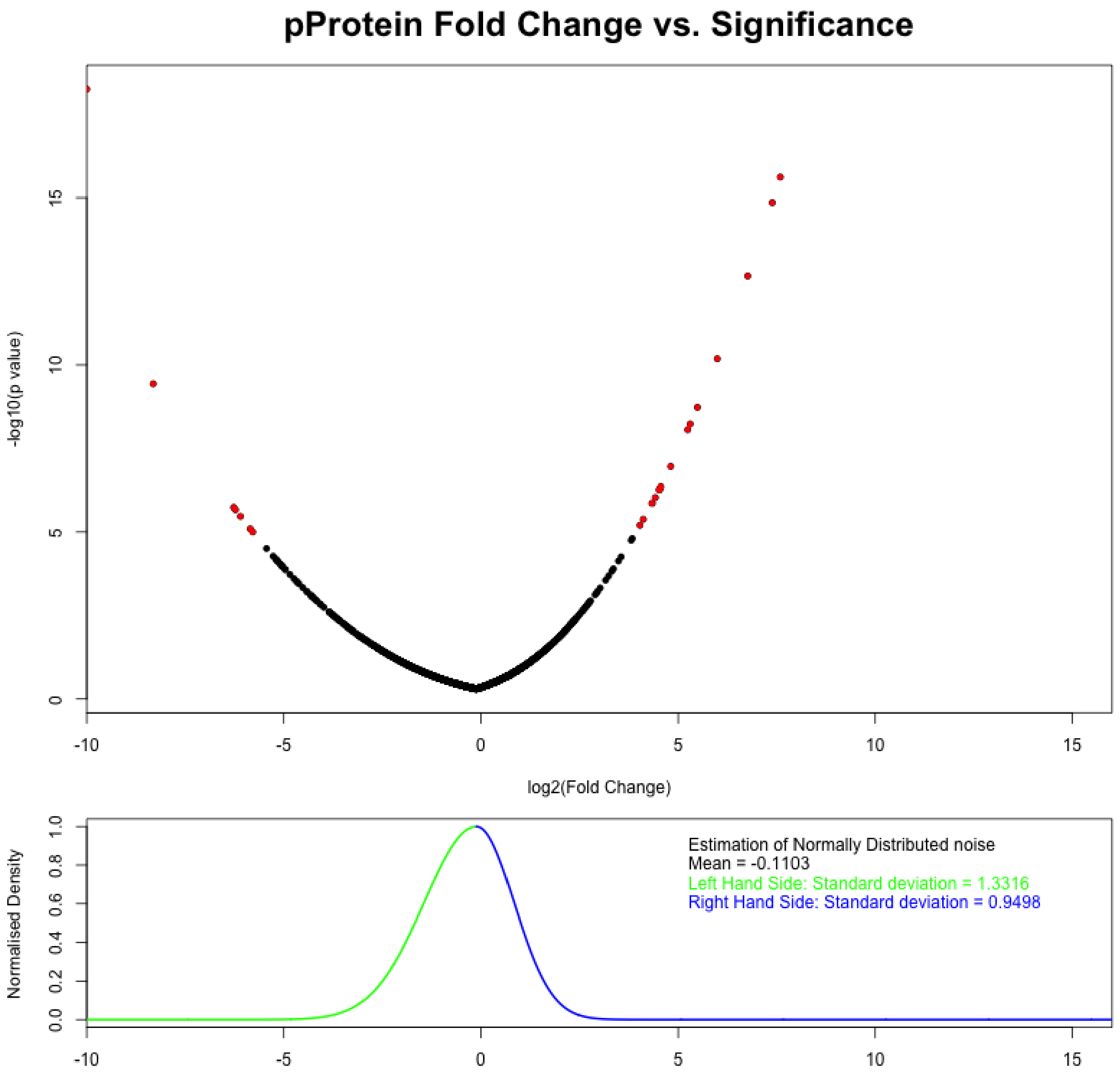
pProtein Fold Change vs. Significance
-log10(p value)
log2(Fold Change)
Normalised Density
Estimation of Normally Distributed noise
Mean = -0.1103
Left Hand Side: Standard deviation = 1.3316
Right Hand Side: Standard deviation = 0.9498

c

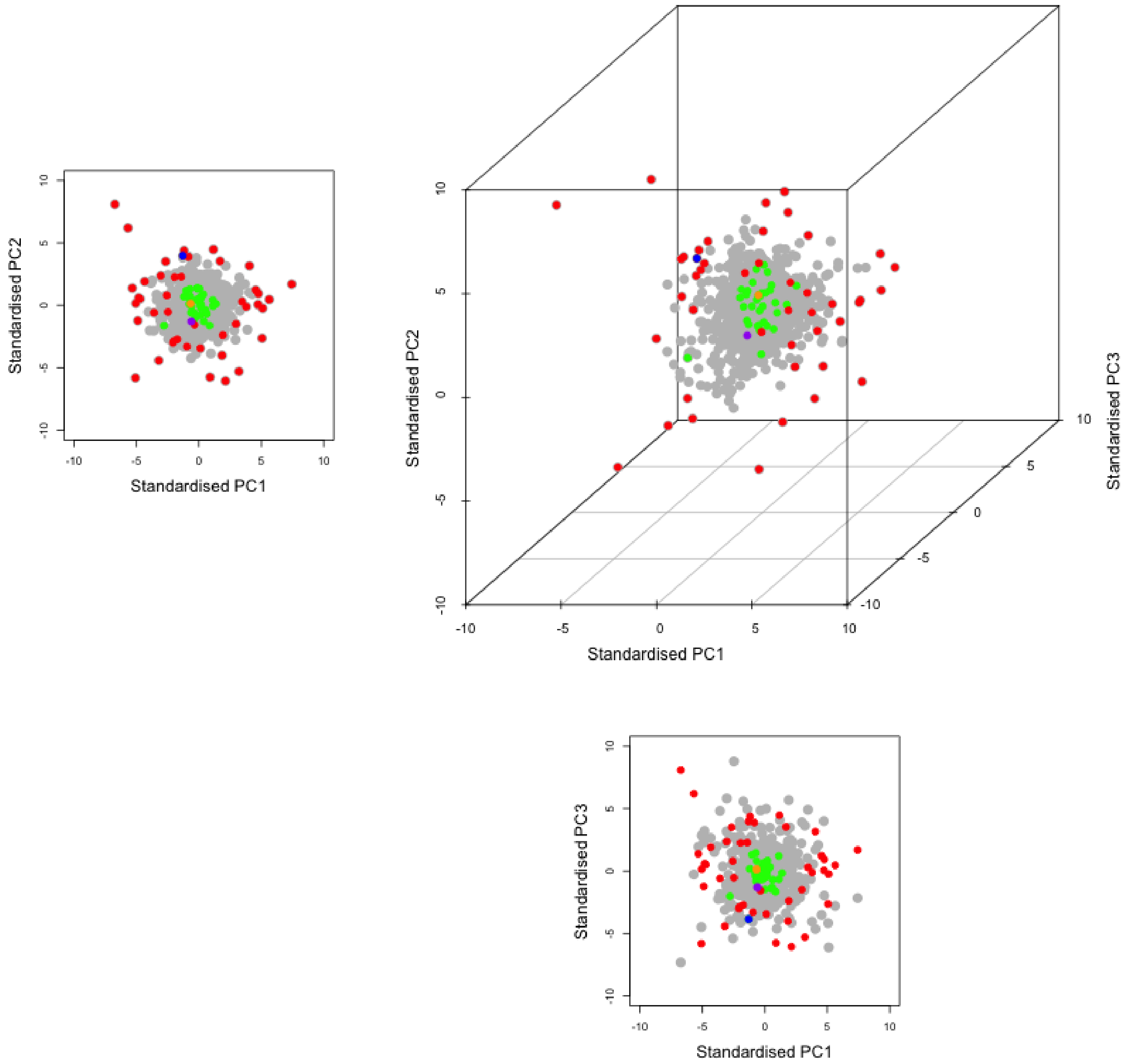
Standardised PC1
Standardised PC2
Standardised PC3
-10
-5
0
5
10

d

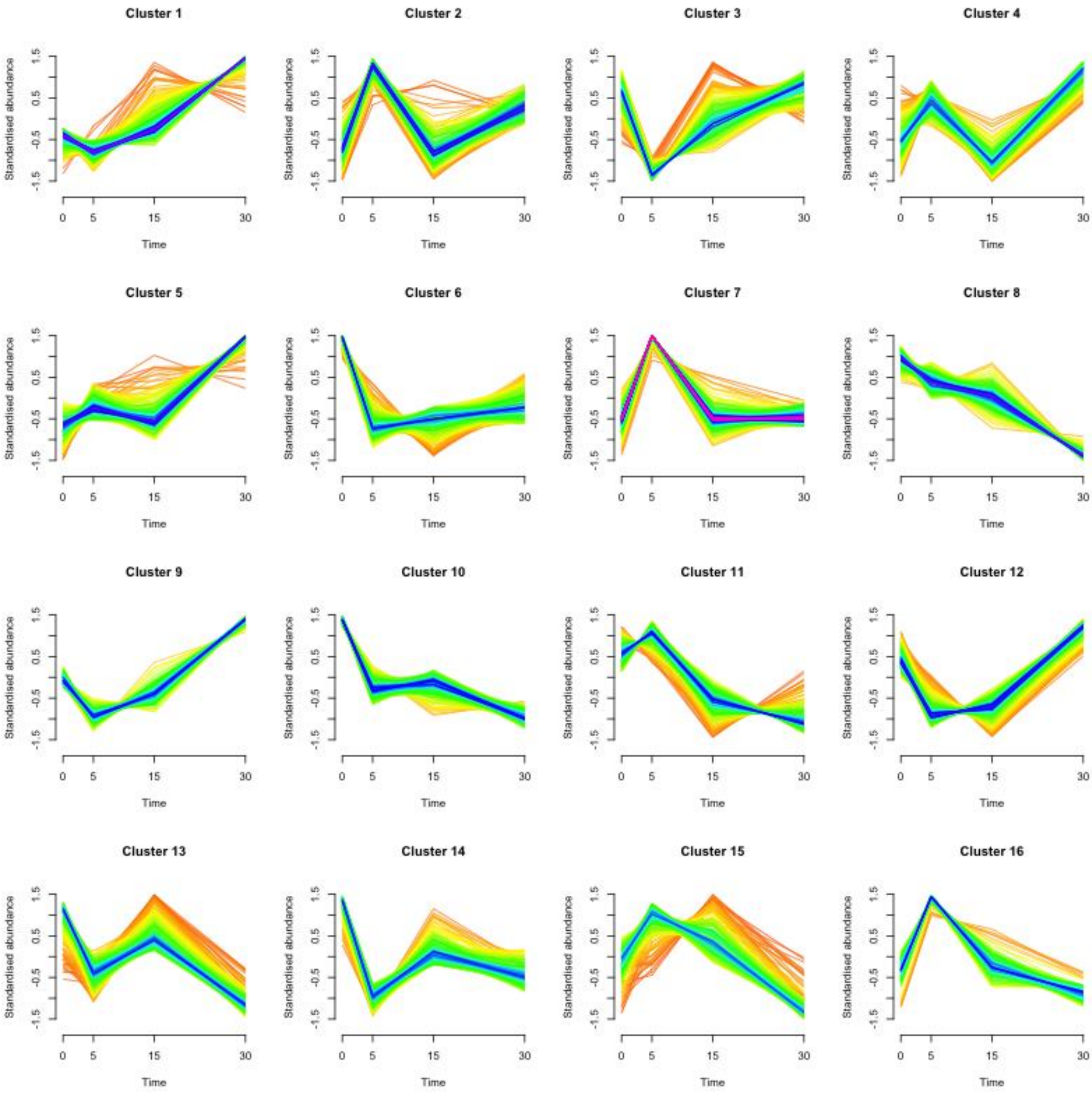
Cluster 1
Cluster 2
Cluster 3
Cluster 4
Cluster 5
Cluster 6
Cluster 7
Cluster 8
Cluster 9
Cluster 10
Cluster 11
Cluster 12
Cluster 13
Cluster 14
Cluster 15
Cluster 16
Standardised abundance
Time

e

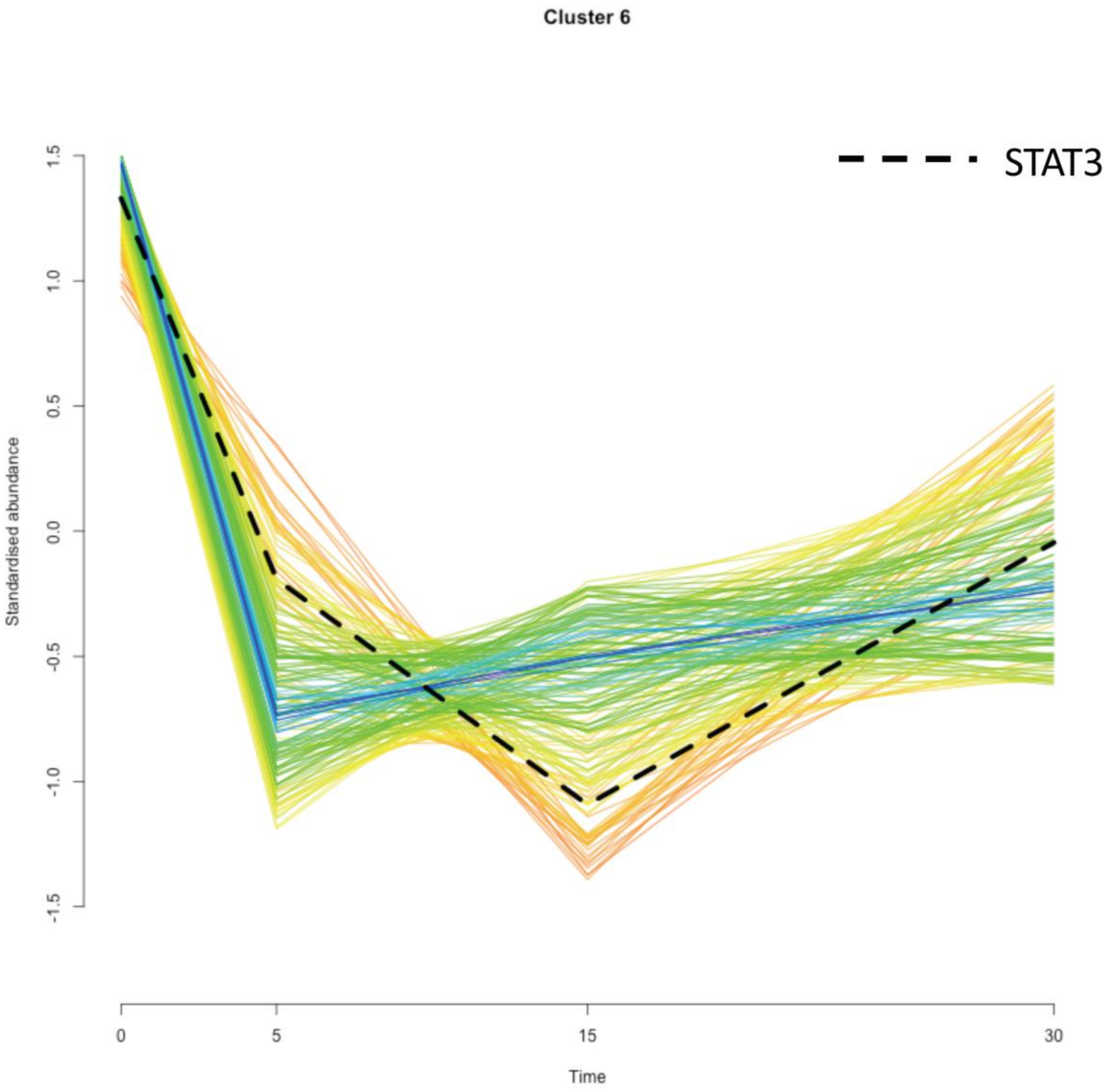

Cluster 6
STAT3
Standardised abundance
1.5
1.0
0.5
0.0
-0.5
-1.0
-1.5
0
5
15
30
Time

f

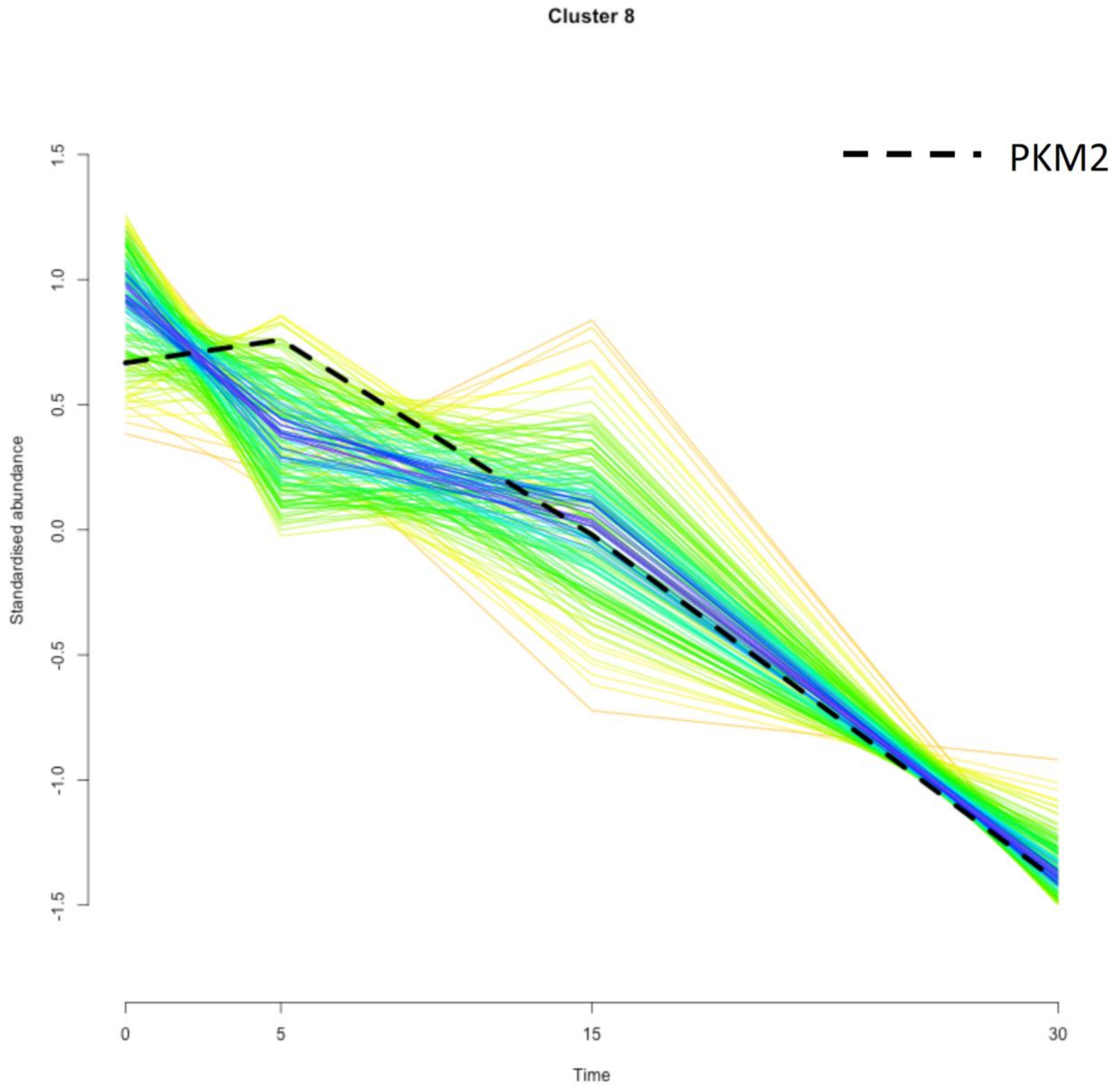

Figure S1. Distribution of quantitative phosphorylation data. (a) (Top panel) Correlation plot of the distribution of phosphopeptide ratios (stimulated/unstimulated). Values are shown as log2 (Fold Change) (x-axis) versus -log10 (p value) (y-axis) in the manner of a standard 'volcano plot'. However, p-values were calculated directly from log2 (Fold Changes) via the complementary error function (after MaxQuant Significance A – see supplemental methods) using separate standard deviation estimators for the lower and upper tails of the underlying

normal error distribution. Points highlighted in red are significant for a false discovery rate of 0.01 using the Benjamini–Hochberg–Yekutieli procedure for controlling error rate. (Bottom panel) Projection of the estimated normal error using the calculated estimators of standard deviation. (b) (Top panel) Correlation plot of the distribution of phosphoprotein ratios (t=30 min/t=0 min). See section A above for description of components. (Bottom panel) Projection of the estimated normal error using the calculated estimators of standard deviation. (c) Principal Component Analysis (PCA) of the full phosphoprotein time course (t=0, 5, 15, 30 min). (All panels) Red points: Outliers identified by applying a BHY FDR of 0.01 on p-values determined from the chi-square distribution, using Mahalanobis distance from projection centre as the test statistic. Green points: Proteins found to be significantly changing in the phosphopeptide fold change analysis in A. Blue point: overlap between these two categories - Metalloproteinase inhibitor 1 TIMP1 (P01033). Orange point: STAT3 (P40763). Purple point: PKM2 (P14618). (Left panel) Principal component 1 versus principal component 2. (Upper right panel) Principal component 1 versus principal component 2 versus principal component 3. (Lower right panel) Principal component 1 versus principal component 3. (d) Clusters of phosphoprotein time courses obtained by soft clustering. Individual time-courses coloured by distance from cluster core, tending towards red as distance increases. (e) STAT3-containing cluster from D highlighting STAT3 time-course (black dotted line). (f) PKM2-containing cluster from D highlighting PKM2 time-course (black dotted line).

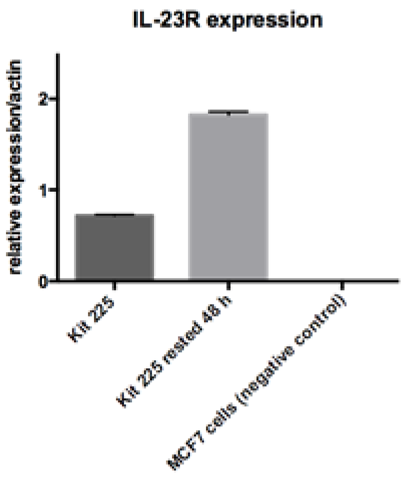

Figure S2. IL-23R expression in Kit223 cells. Relative IL-23R mRNA levels normalised to beta actin (delta Ct values) in Kit225 cells cultured in IL-2 (Kit225 cells) and Kit225 cells rested for 48 h in medium without IL-2. MCF7 cDNA was used as negative control. Data shown are from n = 2 independent biological replicates.

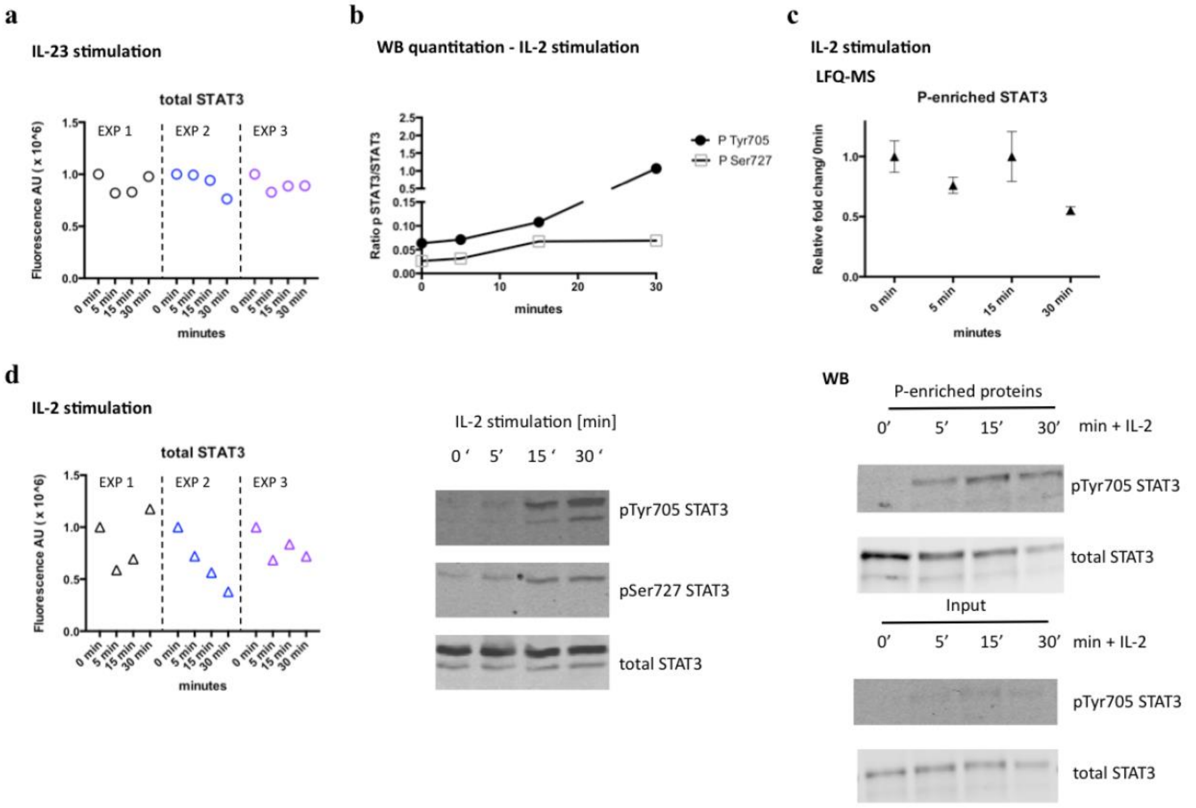

Figure S3. Activation of STAT3 signaling in Kit225 cells stimulated with IL-23 and IL-2. (a) Quantitation of total STAT3 levels in phospho-enriched protein fractions by Western Blot in response to IL-23 stimulation for the indicated times, datasets from 3 biological replicates (EXP1-3) are shown. (b) Kinetics of Tyr705 and Ser727 STAT3 phosphorylation in response to IL-2 stimulation, (c) Levels of total STAT3 detected in phospho-enriched protein fractions after IL-2 stimulation measured by label free quantitation MS (LFQ-MS, top panel) and pTyr05 STAT3 and total STAT3 levels detected by Western Blot (bottom panel, P-enriched proteins, representative Blot experiment 2). (d) Quantitation of total STAT3 levels in phospho-enriched protein fractions by Western Blot in response to IL-23 stimulation for the indicated times, datasets from 3 biological replicates (EXP1-3) are shown.

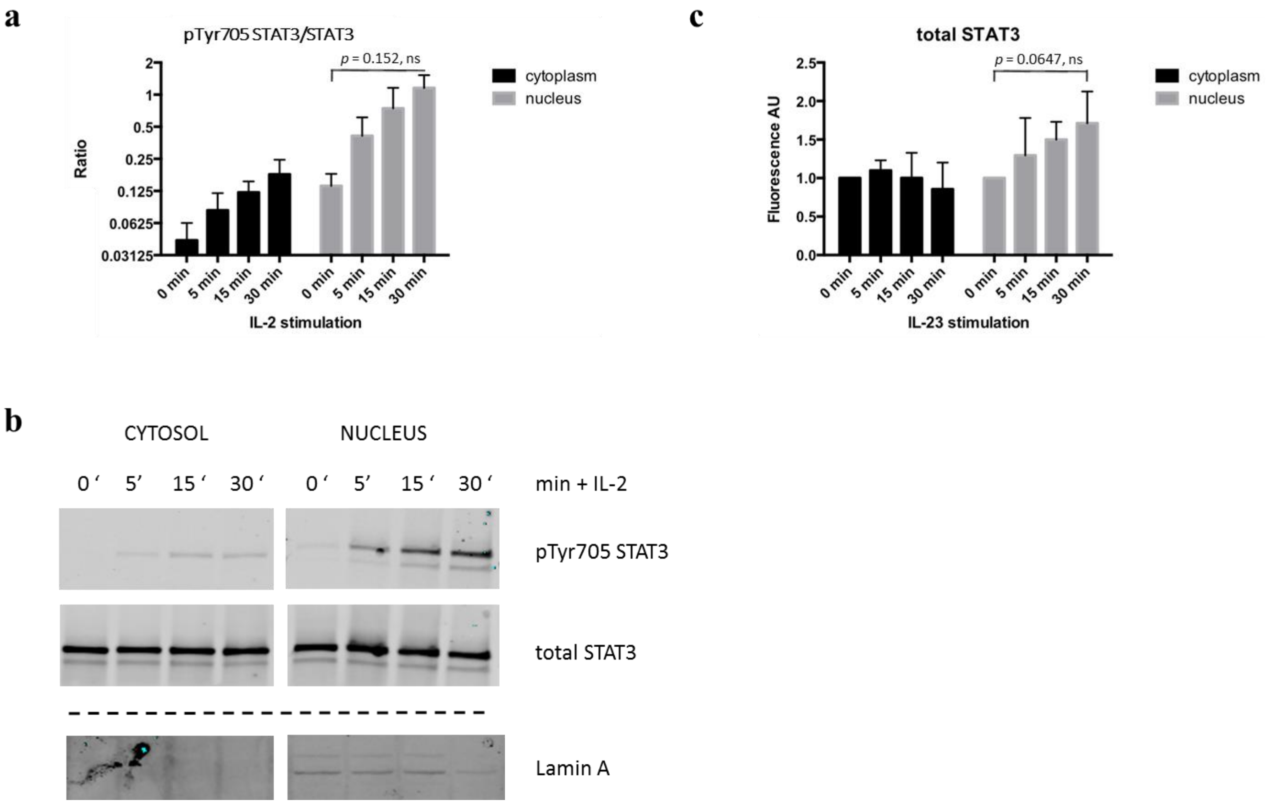

Figure S4. Detection of STAT3 phosphorylation after IL-2 stimulation and monitoring of total STAT3 protein levels in response to IL-23 stimulation. (a) Quantitation of pTyr705 STAT3/STAT3 levels by Western Blot in cytoplasm (black) and nuclear enriched fraction (grey) of IL-2 stimulated Kit225 cells, n=3; p value indicated for comparison of nuclear pTyr705 STAT3/STAT3 levels between 0 and 30 minutes (paired t test using log-transformed data). (b) Representative Western Blot of phosphorylated and total STAT3 in cytoplasmic and nuclear enriched protein fraction after IL-2 stimulation; representative Lamin A blot of cytosolic and nuclear enriched fraction shown (bottom) (c) Quantitation of total STAT3 levels in cytosol and nucleus in cells stimulated with IL-23; p value indicated for comparison of nuclear STAT3 levels at 0 and 30 minutes (paired t test using log-transformed data).

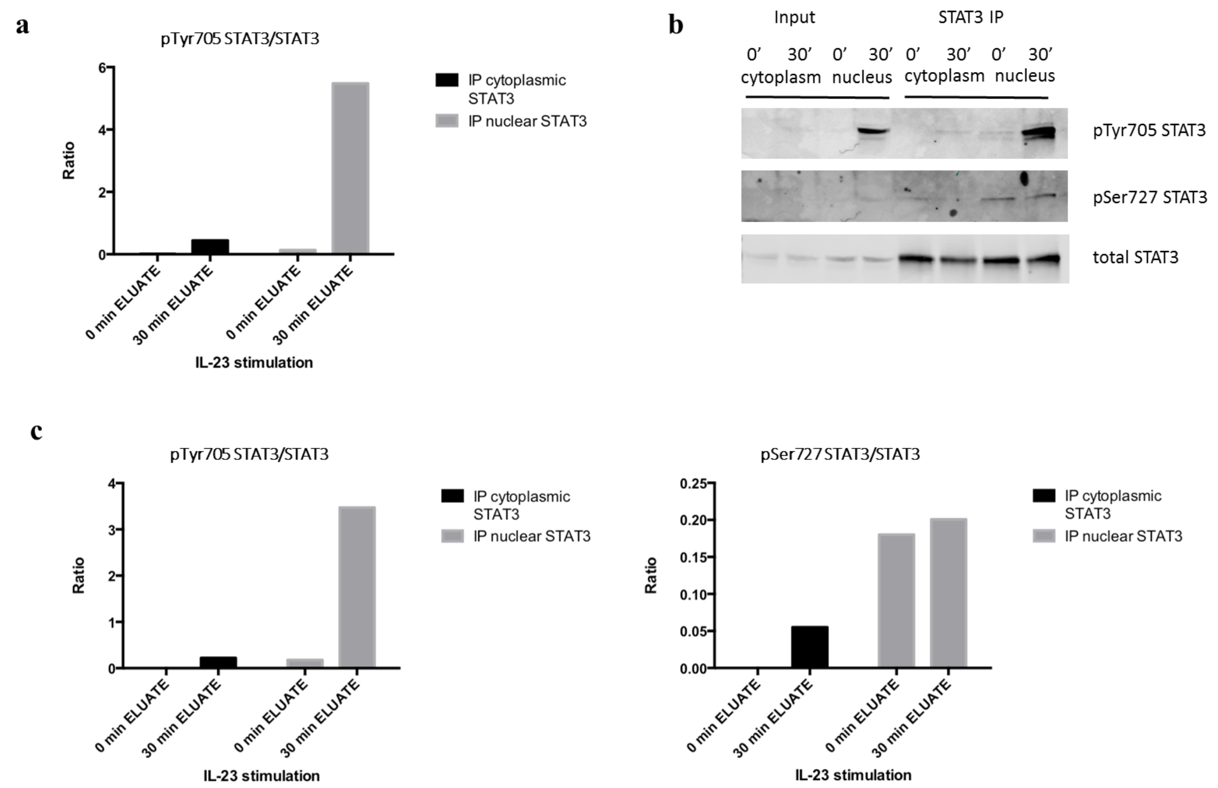

Figure S5. STAT3 phosphorylation in the cytosol and nucleus. (a) Quantitation pTyr705 STAT3/STAT3 levels by Western Blot in cytosolic and nuclear enriched fractions after STAT3 IP. (b) Western Blot for phosphorylated and total STAT3 of cytosolic and nuclear fractions before and after STAT3 IP for samples stimulated with IL-23 (30 min) and unstimulated samples (0 min) (c) Comparison of pTyr705 and pSer727 STAT3 levels in immunoprecipitated cytosolic and nuclear enriched fractions.

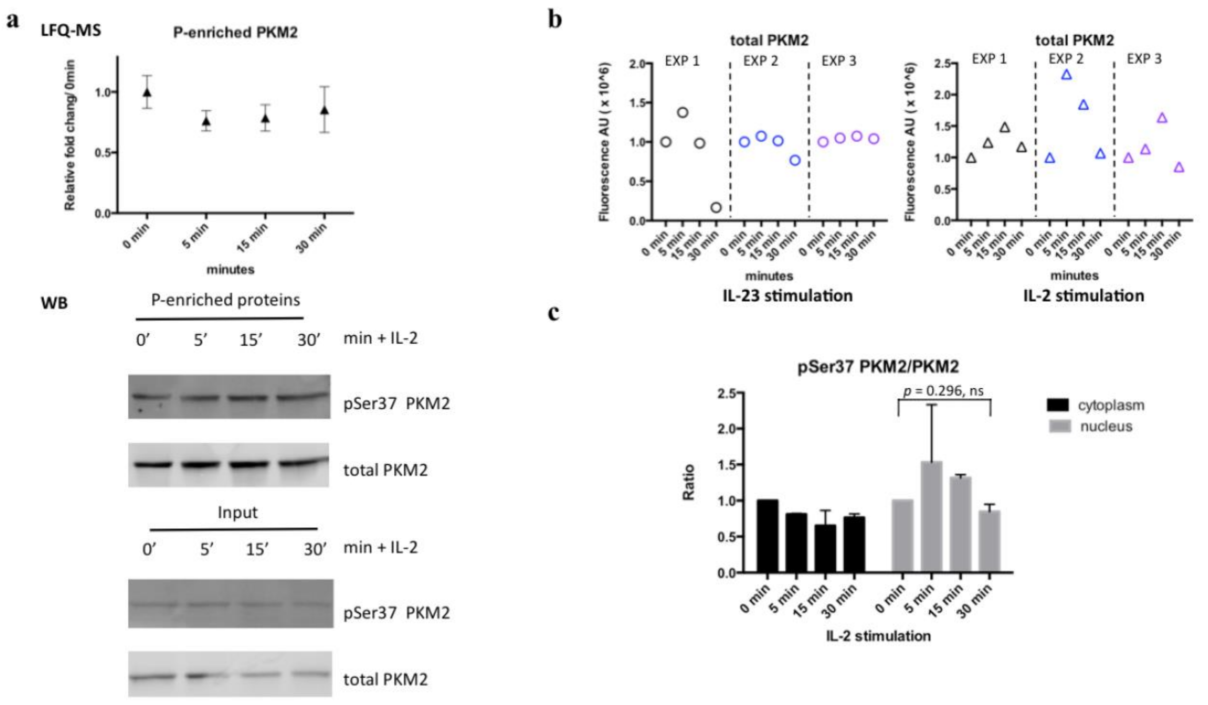

Figure S6. Phosphorylation of PKM2 after IL-23 or IL-2 stimulation. (a) Levels of total PKM2 detected in phospho-enriched protein fractions after IL-2 stimulation measured by label free quantitation MS (LFQ-MS, top panel) and pSer37 PKM2 and total PKM2 levels detected by Western Blot, representative Blot experiment 1 (bottom panel, P-enriched proteins), (b) Quantitation of total PKM2 levels in phospho-enriched protein fractions by Western Blot in response to IL-23 (left) and IL-2 (right) stimulation for the indicated times, datasets from 3 biological replicates (EXP1-3) are shown. (c) Quantitation of pSer37 PKM2/PKM2 levels by Western Blot in cytoplasm (black) and nuclear enriched fraction (grey) in response to IL-2 stimulation, n=2; p value indicated for comparison of nuclear pSer37 PKM2/PKM2 levels between 0 and 30 minutes (paired t test using log-transformed data).

		Precursor ion intensities (averaged EIC[a])			After applying 4.3 correction factor to pY705	
		**Ctrl**	**+IL23**		**Ctrl**	**+IL23**
**pY705**	nucleus	1[b]	5,914,165		4.3	25,405,921
	cytosol	1[b]	226,726		4.3	973,963
	Sum	2	6,140,891		8.6	26,379,884
	ΔpY705		6,140,889	(x 4.3) →		26,379,876
**Y705**	nucleus	27,881,420	17,165,434			
	cytosol	39,811,238	24,147,348			
	Sum	67,692,658	41,312,782			
	ΔY705	26,379,876				
Correction factor for pY705 intensity		=		ΔY705 / ΔpY705 = 4.3		

[a]Extracted ion chromatogram
[b]Inferred as no real MS peak detected

Figure S7. MS quantitation of STAT3 phosphorylation at Tyr705 after IL-23 stimulation. Mass spectrometry (MS) based measurements of STAT3 pTyr705 phosphorylation as described in Figure 5b allowed the exact calculation of Tyr705 and pTyr705 occupancy, as depicted in Figure 5c. A correction factor of 4.3 is applied to balance loss of Tyr705 and gain of pTyr705 signal.

SUPPLEMENTAL TABLES

Table S1. Phosphopeptides identified by mass spectrometry**.** i) A summary table of all phosphopeptides detected by IMAC and TiO2 based phosphopeptide enrichment strategies in datasets A, B, C, the specific sites identified therein, their associated proteins and position within that protein. (In cases where Proteome Discoverer protein grouping assigned a different master protein for a peptide in different analyses, the majority consensus was used) [“Peptide Summary” tab]. ii) Original Proteome Discoverer exported results lists for phosphopeptide enrichment [“Orig PD” tabs]. iii) Final filtered versions of the Proteome Discoverer exported results lists [“Final” tabs]. The Original (ii) and Filtered (iii) tabs contain experiment-level phosphorylation site and isoform probabilities, as calculated by PhosphoRS.

Table S2. Phosphoproteins identified by mass spectrometry**.** Complete list of phosphoproteins identified with 1% FDR (quantitation and normalisation based on peptides identified with a Mascot score >20) in Kit225 cells stimulated for 0, 5, 15, 30 min with IL-23. Label free quantitation and data analysis with Progenesis.

Table S3. IL-23 altered downstream phosphoprotein targets with overlapping phosphosites identified. Proteins were selected that showed phosphopeptides with >= 1.5 fold and protein levels with > 2-fold change (upregulated and downregulated proteins are in separate labelled tabs). The columns shown are: Phosphopeptides with corresponding protein accession number (UniProt); protein description; peptide start/end; phosphosites identified; phosphopeptide fold change in normalised HILIC/IMAC datasets A, B, C and mean and

TiO2 datasets A, B and mean; mean (n=2) phosphoprotein fold change at t = 0, 5, 15, 30 minutes.

SUPPLEMENTAL METHODS

Cell lines and antibodies

Kit225 cells were grown in RPMI/10 % FCS/PS + 2 ng/ml rhIL-2 (Proleukin, Novartis Pharmaceuticals UK). Kit225 cDNA was prepared using single strand cDNA Kit (Invitrogen). MCF7 cDNA was a gift from Dr. David Barnes (NDORMS, University of Oxford). IL-23R and beta actin mRNA levels were determined by RT-PCR using TaqMan probes (probes Hs0032759 and HS99999903, Applied Biosystems). Antibodies used were: rabbit anti-pTyr705 STAT3 (D3A7) 1:1000 (Cell Signaling #9145), rabbit anti-pSer727 STAT3 1:1000 (Cell Signaling #9134), mouse anti-STAT3 (124H6) 1:1000 (Cell Signaling #9139), rabbit anti-pSer37 PKM2 1:1000 (Biorbyt orb76553), rabbit anti-PKM2 1:2000 [EPR10138(B)] (Abcam ab150377), rabbit anti-lamin A 1:1000 (Biolegend #613502), goat anti-rabbit IgG (H+L) IRDye 800CW 1:10000 (LICOR Biosciences), goat anti-mouse IgG (H+L) IRDye 680RD 1:10000 (LICOR Biosciences).

Analysis by tandem mass spectrometry

Dried peptide samples were resuspended in 2 % acetonitrile and 0.1 % trifluoroacetic acid and analyzed by nano–liquid chromatography tandem mass spectrometry (nano–LC-MS/MS) as described previously (1). In brief, samples were separated using a nanoUPLC (Easy spray C18 column with a 75 µm × 500 mm, 2.1 µm particle size; Thermo-Fisher) coupled to a Q Exactive tandem mass spectrometer (Thermo Scientific, Bremen, Germany). MS data was acquired with a resolution of 70000 at m/z 200 and selecting the Top 15 precursor ions. Ion target in MS1 was $3x10^6$ and $5x10^5$ in MS2 mode. Ions were accumulated for up to 100 ms in

MS1 and 128 ms in MS2. The samples were loaded in 0.1% TFA in 1% ACN. The gradient used to elute the peptides started by a 3 minute isocratic gradient composed with 3% buffer B (0.1% FA in CH_3CN) followed by a linear gradient from 3–35% of buffer B for 60 minutes at a flow rate of 250 nl/minute and a two washes with 97% of buffer B for 3 minutes. The total length of the analysis was 100 min to allow column re-equilibration.

Data analysis

Phosphopeptide analysis: Results are from three different independent biological replicates comparing unstimulated Kit225 cells (t = 0 min) versus Kit225 cells after 30 minutes stimulation with IL-23 (t = 30 min). Cells within one experiment were taken from the same culture. Cells for experiments A and B were from the same batch and equal passage number, cells for experiment C were a separate batch with same passage number. All batches originated from cells frozen on the same date and stored in liquid nitrogen. Unstimulated/stimulated samples were digested as described above and the peptides labelled with two different dimethyl labels per the table below; note the inverted label strategy between dataset A and datasets B and C.

Table S4. Experimental design of the phosphopeptide based proteomics approach used in this study.

Dataset	Unstimulated (t = 0) Label	Stimulated (t = 30) Label	Cell Numbers/Condition
A	2H4 Dimethyl	Dimethyl	100×10^6
B	Dimethyl	2H4 Dimethyl	100×10^6
C	Dimethyl	2H4 Dimethyl	50×10^6

Analysis of the MS data for these phosphopeptide datasets was performed using Proteome Discoverer v1.4 (PD, Thermo Scientific) and the default Dimethylation 2plex method. As search node we used Mascot (v2.3) and the Uniprot Swissprot database (human, retrieved 08/12/2013) with the following parameters: Missed cleavage = 1, Peptide Cut Off Score = 10, Precursor Mass Tolerance = 10 ppm, Fragment Mass Tolerance = 0.02 Da, Dynamic Modifications = Oxidation(M); Dimethyl/Dimethyl 2H(4) (K, N-term);Phospho (ST,Y), Static Modifications = Carbamidomethyl (C), Percolator Maximum Delta Cn = 0.05 & Target FDR = 0.05). Precursor quantitation settings were Mass Precision = 3 ppm, S/N Threshold = 1, RT Tolerance of Isotope Pattern Multiplets = 1 min, Single-Peak/Missing Channels Allowed = 1, Replace Missing Quan Values With Minimum Intensity = True. The results were initially filtered in PD according to the following parameters - quantify unique peptides only, Maximum Fold Change = 10000, Minimum Quantitation Value threshold = 0.1, Mascot cut-off value = 20, false discovery rate = 1 %, Peptide Grouping disabled, Protein Grouping enabled (using Strict Maximum Parsimony Principle). The list was refined to select only phosphopeptides, exported as .csv and further filtered in Excel to select Search Rank = 1, Unambiguous (allowing for protein grouping) peptides. For each phosphopeptide, in cases of multiple data points due to multi-modal elution profiles or different charge states, the example with the highest mean abundance across both conditions was taken. These final quantitation values for unique phosphopeptides are shown in the filtered data tabs of Table S1. Finally, stimulated/unstimulated ratios were normalized to correct for mixing error by dividing all values by the median of all peptides exported from PD (normalization factor for datasets: A: 1.10, B: 1.14, C: 0.95).

Statistical analysis of differential phosphorylation at the peptide & protein level

We first compared the phosphopeptide and the phosphoprotein datasets like-for-like, calculating the log2 fold change for 30 min versus 0 min. In order to apply a consistent method of quantitation thresholding for both the IMAC phosphopeptide and the phosphoprotein datasets (30 versus 0 min IL-23 stimulation), we used the 'significance A' p-value described for the MaxQuant quantitation package (2). In brief, for each measured changed (phosphopeptide or phosphoprotein), we computed the probability of obtaining a log-ratio of at least that magnitude under the null hypothesis that the distribution of log-ratios had normal upper and lower tails (Figure S1a, b). As per the MaxQuant method, separate estimators of standard deviation were used for the upper and lower tails to allow thresholding to be more robust in the face of bias toward up- or down-regulation. We defined as significant any points with a false discovery rate <= 0.01, calculated from the p-values using the Benjamini–Hochberg–Yekutieli (BHY) procedure for controlling error rate / correction for multiple testing (3). Additional statistical analysis was performed taking into account the full phosphoprotein dataset that consisted of a time-course with data points at 0, 5, 15 and 30 min. Log2-fold changes were standardized (normalized to zero mean and unit variance). We first analyzed variance in the data set by PCA, marking outliers based on BHY-corrected chi-square p-values based on the Mahalanobis distance from the center of the projection (Figure S1c) (4, 5). Secondly, to cluster phosphoproteins by similarity across the whole time course, we used the Mfuzz soft clustering package in R (6) based on its previous deployment in phosphoprotein time-course analysis (7). The included 'mestimate' function was used to calculate the fuzzifier parameter (8), and a cluster number parameter of 16 was selected from a range of target cluster numbers from 4 through 40 as the point at which the minimum centroid distance for each cluster number moved from rapid decline to slow decline. Clustering was repeated for 100 iterations and the iteration with the highest group membership score for STAT3 was selected (Figure S1d-f).

Subcellular fractionation, immunoprecipitation and immunoblotting

Kit225 cells were stimulated with IL-23 or IL-2 for 5, 15 and 30 min (5 Mio. cells/time point in 1 ml RPMI, including unstimulated control sample) and lysed with the buffer (120 µl) used for phosphoprotein enrichment as described above. After centrifugation at 3000 rpm, cytosolic fraction was retrieved and nuclei lysed for 30 min on ice in 120 µl 40 mM TrisHCl, 0.4 M NaCl, 2 mM EDTA. 2.5 mM $MgCl_2$, 1 % NP-40, 0.1 % SDS, 1 % deoxycholate, 125 U/ml Benzonase (Merck Millipore) and protease and phosphatase inhibitors (see above). Insoluble material was pelleted by centrifugation at 10000 rpm, 10 min, 4 °C and nuclear extract was stored. Protein concentrations for both fractions were determined and equal amounts per sample used for subsequent Western Blot analysis.

For immunoprecipitation of STAT3, 50 x 10E6 cells per time point were used and stimulated with IL-23 as described above (30 min) or left unstimulated (0 min). For preparation of cytosolic and nuclear fractions, cells were lysed in 1 ml lysis buffer used for phosphoprotein enrichment containing protease and phosphatase inhibitors and after retrieving cytosolic fraction nuclei were lysed in 1 ml nuclear lysis buffer + protease and phosphatase inhibitors and insoluble material was cleared as described above. After removing an aliquot of the lysate for Western Blot analysis, the resulting 2 cytosolic fractions and 2 nuclear fractions were diluted 10-fold with NET buffer (50 mM TrisHCl, 5 mM NaCl, 5 mM EDTA, 0.5 % NP40) and processed in parallel. Protein G agarose (Life Technologies) was washed in NET buffer before being used. Lysates were pre-cleared by addition of 125 µl of settled Protein G agarose beads for 30 min, 4 °C followed by incubation with 25 µl anti-total STAT3 antibody for 40 min, 4 °C and adding 125 µl Protein G agarose for another 30 min.

The resin was then washed 3 x with NET buffer and proteins eluted with 100 mM glycine, pH 2.5. After removing an aliquot for Western Blot analysis, samples were chloroform methanol precipitated, digested as before and analyzed by LC-MS/MS.

For Western Blot analysis, samples were run on Criterion XT Bis-Tris Precast Gels (Bio-Rad) and blotted onto Odyssey 0.22 µm nitrocellulose membranes (LICOR Biosciences). Primary and secondary antibodies were prepared in Odyssey Blocking buffer (LICOR Biosciences) and washing steps were performed using 1 x PBS/0.05 % Tween-20. Phosphospecific and total antibodies were incubated 1 h RT in sequential steps followed by incubation with IRDye labelled secondary antibodies for 1 h, RT, with three washes between. When using 2 antibodies generated in the same species, phosphospecific and secondary antibody were stripped off with ReBlot Plus strong antibody stripping solution (Millipore) before blocking and incubation with total antibody and respective secondary antibody as described. Blots were scanned using the Odyssey CLx infrared imaging system and band intensities analyzed with the Image Studio software (LICOR Biosciences).

Gene expression analysis

For gene expression analysis, 5 x 10^6 cells were rested as described above, resuspended in full medium at a cell density of 1 x 10^6 cells/ml and either left unstimulated or stimulated with IL-23 or IL-2 (10 ng and 20 ng (360 U)/5 x 10^6 cells). After 24 h RNA was isolated using RNeasy Mini Kit (Qiagen) and reverse transcribed using a mix of oligo(dT) and random pentadecamer primers followed by amplification of target genes using unlabelled primers and SYBR® Green RT PCR Kit (Applied Biosystems). Primers were designed to span exon intron barriers and specificity of product amplification ensured by measuring melting curves. Gene expression data were analysed using the Delta C(t) method (9).

Statistics applied was ratio paired t test using log transformed data with $p < 0.05$ being statistically significant.

Lactate measurement by GCxGC-MS

For intracellular lactate measurement 2.5 x 10^6 rested cells were left unstimulated or stimulated either with IL-23 (see above) or with Dynabeads coated with αCD3/αCD28 beads (Invitrogen, 1.25 beads per cell) for 24 h. There was no difference in cell numbers between the different conditions (unstimulated versus stimulated) after 24 h. Extraction of metabolites was performed by treating cells with 400µl methanol/H_2O (1:1) and homogenization using washed glass beads in a bead beater (Precellys 24, Bertin Technologies) for four cycles (6500 Hz, 45s), followed by the addition of 1 ml tert-butyl ether (MTBE) to extract metabolites. After vortexing for 5 min and centrifugation for 20 min at 13000 g and 4°C, the organic phase was transferred to a glass vial and dried under vacuum. To the remaining aqueous phase, 800µl of methanol was added, samples vortexed for one cycle (6500 Hz, 45s), kept at -80°C for one hour and centrifuged for 20 min at 13000 g and 4°C. 1ml of aqueous phase was removed, added to the glass bead vial containing the organic phase and the samples dried under vacuum. Chemical derivatization was performed essentially as described (10). In brief, samples were resuspended in 50µl of a 20µg/µl solution of methoxyamine hydrochloride in pyridine and incubated for 90 min at 30°C and shaking (1200 rpm). 70µl of N-Methyl-N-trimethylsilyltrifluoroacetamide (MSTFA) with 1% chlorotrimethylsilane (TMCS) were added to the samples, followed by incubation for one hour at 60°C and shaking (1200 rpm). The samples were immediately analysed using a GCxGC-MS system comprising of a gas chromatograph coupled to a quadrupole mass spectrometer (Shimadzu GCMS QP2010 Ultra)

and a Shimadzu AOC-20i/s auto sampler as described (11). Raw GCxGC MS data was processed using GCMSsolution software (Shimadzu), and Chromsquare software (V1, Shimadzu) in combination with the NIST11/s, OA_TMS, FA_ME and YUTDI libraries were used for data analysis and annotation of lactate. Technical triplicates were analysed using an unpaired Student's T test.

SUPPLEMENTAL REFERENCES

1. Fischer, R.; Kessler, B. M., Gel-aided sample preparation (GASP)-A simplified method for gel-assisted proteomic sample generation from protein extracts and intact cells. *Proteomics* **2015,** 15, (7), 1224-9.
2. Cox, J.; Mann, M., MaxQuant enables high peptide identification rates, individualized p.p.b.-range mass accuracies and proteome-wide protein quantification. *Nat Biotechnol* **2008,** 26, (12), 1367-72.
3. Benjamini, Y.; Drai, D.; Elmer, G.; Kafkafi, N.; Golani, I., Controlling the false discovery rate in behavior genetics research. *Behav Brain Res* **2001,** 125, (1-2), 279-84.
4. Brereton, R. G., The chi squared and multinormal distributions. *Journal of Chemometrics* **2015,** 29, 9-12.
5. Brereton, R. G., The Mahalanobis distance and its relationship to principal component scores. *Journal of Chemometrics* **2015,** 29, 143-145.
6. Kumar, L.; E Futschik, M., Mfuzz: a software package for soft clustering of microarray data. *Bioinformation* **2007,** 2, 5-7.
7. Olsen, J. V.; Blagoev, B.; Gnad, F.; Macek, B.; Kumar, C.; Mortensen, P.; Mann, M., Global, in vivo, and site-specific phosphorylation dynamics in signaling networks. *Cell* **2006,** 127, 635-48.
8. Schwämmle, V.; Jensen, O. N., A simple and fast method to determine the parameters for fuzzy c-means cluster analysis. *Bioinformatics (Oxford, England)* **2010,** 26, 2841-8.
9. Livak, K. J.; Schmittgen, T. D., Analysis of relative gene expression data using real-time quantitative PCR and the 2(T)(-Delta Delta C) method. *Methods* **2001,** 25, (4), 402-408.
10. Marney, L. C.; Kolwicz, S. C.; Tian, R.; Synovec, R. E., Sample preparation methodology for mouse heart metabolomics using comprehensive two-dimensional gas chromatography coupled with time-of-flight mass spectrometry. *Talanta* **2013,** 108, 123-130.
11. Weinert, C. H.; Egert, B.; Kulling, S. E., On the applicability of comprehensive two-dimensional gas chromatography combined with a fast-scanning quadrupole mass spectrometer for untargeted large-scale metabolomics. *Journal of Chromatography A* **2015,** 1405, 156-167.
